# Effects of methionine deficiency on B7H3-DAP12-CAR-T cells in the treatment of lung squamous cell carcinoma

**DOI:** 10.1038/s41419-023-06376-w

**Published:** 2024-01-05

**Authors:** Tao Yu, Feng-Qi Nie, Qi Zhang, Shao-Kun Yu, Mei-Ling Zhang, Qian Wang, En-Xiu Wang, Kai-Hua Lu, Ming Sun

**Affiliations:** 1 Department of Oncology, The First Affiliated Hospital of Nanjing Medical University, No. 300 Guangzhou Road, Nanjing, China; 2 Department of Oncology, The Second Affiliated Hospital, Nanjing Medical University, Nanjing, China; 3 Department of Oncology, The Affiliated Taizhou People’s Hospital of Nanjing Medical University, Taizhou, China; 4 Nanjing CART Medical Technology Co., Ltd, Nanjing, China; 5 Suzhou Cancer Center Core Laboratory, Suzhou Municipal Hospital, Gusu School, The Affiliated Suzhou Hospital of Nanjing Medical University, Suzhou, China

**Keywords:** Non-small-cell lung cancer, Tumour immunology, Cancer metabolism

## Abstract

Lung squamous cell carcinoma (LUSC) is a subtype of lung cancer for which precision therapy is lacking. Chimeric antigen receptor T-cells (CAR-T) have the potential to eliminate cancer cells by targeting specific antigens. However, the tumor microenvironment (TME), characterized by abnormal metabolism could inhibit CAR-T function. Therefore, the aim of this study was to improve CAR-T efficacy in solid TME by investigating the effects of amino acid metabolism. We found that B7H3 was highly expressed in LUSC and developed DAP12-CAR-T targeting B7H3 based on our previous findings. When co-cultured with B7H3-overexpressing LUSC cells, B7H3-DAP12-CAR-T showed significant cell killing effects and released cytokines including IFN-γ and IL-2. However, LUSC cells consumed methionine (Met) in a competitive manner to induce a Met deficiency. CAR-T showed suppressed cell killing capacity, reduced cytokine release and less central memory T phenotype in medium with lower Met, while the exhaustion markers were up-regulated. Furthermore, the gene NKG7, responsible for T cell cytotoxicity, was downregulated in CAR-T cells at low Met concentration due to a decrease in m5C modification. NKG7 overexpression could partially restore the cytotoxicity of CAR-T in low Met. In addition, the anti-tumor efficacy of CAR-T was significantly enhanced when co-cultured with SLC7A5 knockdown LUSC cells at low Met concentration. In conclusion, B7H3 is a prospective target for LUSC, and B7H3-DAP12-CAR-T cells are promising for LUSC treatment. Maintaining Met levels in CAR-T may help overcome TME suppression and improve its clinical application potential.

## Introduction

Lung cancer is the leading cause of tumor mortality, posing a serious threat to public health [1]. Non-small cell lung cancer (NSCLC) is the predominant histological type of lung cancer, including lung adenocarcinoma (LUAD) and lung squamous cell carcinoma (LUSC). It is noteworthy that targeted drugs significantly prolonged the survival of patients with LUAD with frequent driver mutations [2]. Meanwhile, immunotherapy has become an important part of regimens for lung cancer [3]. However, the treatment of LUSC remains challenging due to the low mutation frequency of driver genes in LUSC [4]. Only around 20% of patients with LUSC can benefit from immune checkpoint inhibitors (ICIs) due to the limited number of immunotherapeutic markers. High TMB in LUSC may even conversely predict poor response to ICI. Additionally, anti-angiogenic agents like bevacizumab increase the hemorrhage risk of LUSC [5]. Given the limited options for first-line treatment of LUSC, we investigated the therapeutical potential of chimeric antigen receptor T-cells (CAR-T) as a potential alternative.

The CAR-T therapy, which utilizes a single-chain variable fragment (scFv) to specifically target tumor cells expressing appropriate antigens, has shown remarkable success in the treatment of hematological cancers [6]. Moreover, more than 100 CAR-T trails focusing on solid tumors are ongoing [7]. However, CAR-T faces several major challenges in solid tumors [8]. First, the physical barriers present in solid tumors can hinder the migration and infiltration of CAR-T cells. Tumor heterogeneity and antigen loss also impede CAR-T recognition. Second, immunosuppressive tumor microenvironment (TME) could induce the exhaustion of CAR-T cells. Third, the majority of NSCLC-associated CAR-T studies have focused on patients with LUAD, ignoring the influence of histological differences between LUAD and LUSC. To address these challenges, CAR-T composed of scFv and stimulation domains must be optimized to adapt to the unique environment of LUSC.

Based on literature review and screening experiments, B7 homolog 3 protein (B7H3) protein was identified as a promising target for LUSC treatment. Similar to PD-L1, B7H3, a member of the B7 protein family, is located on T cell surfaces to regulate T cell functions. B7H3 is expressed at low levels in normal tissues and at high levels in solid tumors, including NSCLC, breast cancer, and liver malignancies [9]. B7H3 contributes to tumor growth by promoting proliferation, invasion, angiogenesis, and abnormal metabolism. High B7H3 was associated with poor prognosis in lung cancer, and its expression was higher in LUSC than in LUAD [10]. Several I/II Phase clinical trials (NCT02475213 and NCT02381314) have evaluated the safety and efficacy of antibody drugs targeting B7H3 [11]. B7H3-targeted CAR-Ts have demonstrated significant cytotoxic effects in animal models, and B7H3 inhibitors have reversed the abnormal tumor metabolism [12], suggesting that reducing B7H3 is beneficial for improving clinical outcomes.

In this study, we confirmed the high expression of B7H3 in LUSC and its cell membrane localization. The CAR-T targeting B7H3 and stimulated by DNAX activating protein 12 (DAP12) was constructed based on our previously developed novel multiple chain DAP-CAR. Our previous studies on leukemia and solid tumors have demonstrated that DAP12 receptor could enhance anti-tumor efficacy by producing massive cytokines [13, 14]. CAR-T with DAP12 showed inspiring outcomes with a low toxicity profile in a Phase I clinical trial. The growth and immune capacities of CAR-T were tested through co-culturing with LUSC cells. This study further investigated the cause and effect of methionine (Met) deficiency in TME on B7H3-DAP12-CAR-T cells.

## Results

### B7H3 is highly expressed in LUSC

**Fig. 1:**
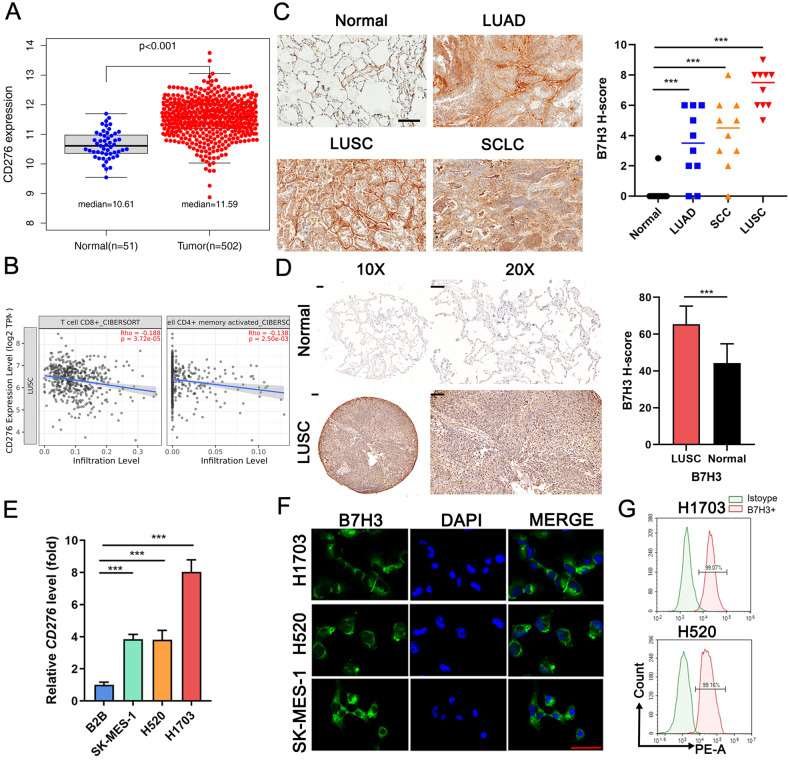
B7H3 is highly expressed in LUSC. **A** Expression of CD276 in LUSC (*n* = 502) and normal samples (*n* = 51) based on TCGA. **B** The relationship between CD276 and CD8 + T cells (R = −0.188, *P* < 0.001) or CD4+ activated memory cells (R = −0.138, *P* < 0.001) in LUSC analyzed by Timer2.0. **C** Histochemistry score (H-score) of B7H3 on 10 samples of normal lung tissues, LUAD, LUSC, and SCLC shown by IHC staining. Scale bars = 200 μm. **D** H-score of B7H3 on 48 pairs of LUSC tissues and adjacent normal tissues shown by IHC. Scale bars = 100 μm. **E** CD276 expressed in SK-MES-1, H520, H1703, and B2B shown by qRT-PCR. **F** B7H3 localization in H1703/H520/SK-MES-1 shown by IF (×40 magnification). Representative images are shown. Scale bars = 100 μm. **G** B7H3 expression on the cell membrane of H1703 and H520 shown by flow cytometry. * *P* < 0.05; ** *P* < 0.01; *** *P* < 0.001. ns, not significant. Variables are presented as mean ± SD.

The Cancer Genome Atlas (TCGA) sequencing data showed that the B7H3 transcript, CD276 was highly expressed in LUSC, while lower in normal tissues (Fig. 1A). In contrast, the PDL1 transcript, CD274 was higher in normal tissues than in lung cancer tissues (Supplementary Fig. S1A). According to GEPIA, the overall survival period of LUSC patients with the top 20% expression of CD276 is shorter than those with the bottom 20% expression. The difference in disease-free survival is more significant (Supplementary Fig. S1B). According to ESTIMATE scores, the expression of CD276 was negatively associated with CD8 + T cells and activated memory CD4 + T cells in LUSC (Fig. 1B). Pan-cancer analysis based on GEPIA also revealed high expression of CD276 in 15 types of malignancies (Supplementary Fig. S1C). A comparison of B7H3 expression was made among 10 specimens each of LUSC, LUAD, small-cell lung cancer (SCLC), and normal tissues. IHC results indicated that B7H3 was highly expressed in LUSC and scarce in normal tissues (Fig. 1C). This was further confirmed by TMA consisting of 48 pairs of LUSC and adjacent lung tissues (Supplementary Table S2), showing that B7H3 was higher in LUSC than in adjacent normal tissues (Fig. 1D). Based on DepMap database, CD276 was highly expressed in 28 LUSC cell lines, and higher than CD274. According to the CRISPR screening data from the DepMap, knockout of CD276 inhibited 22 LUSC cell lines, whereas CD274 depletion did not inhibit their growth (Supplementary Fig. S1D). The higher expression of B7H3 in LUSC cell lines (SK-MES-1, H520, and H1703) than in the pulmonary epithelial cell BEAS-2B (B2B) was confirmed by qRT-PCR (Fig. 1E). IF staining assay showed that B7H3 was abundantly expressed in LUSC cell lines (Fig. 1F). B7H3 expression on the cell membrane of H1703 and H520 was further proved by flow cytometry (Fig. 1G). Thus, B7H3 has the potential to serve as a target of CAR-T therapy.

### B7H3-DAP12-CAR-T exhibits cell-killing effects on LUSC cells

**Fig. 2:**
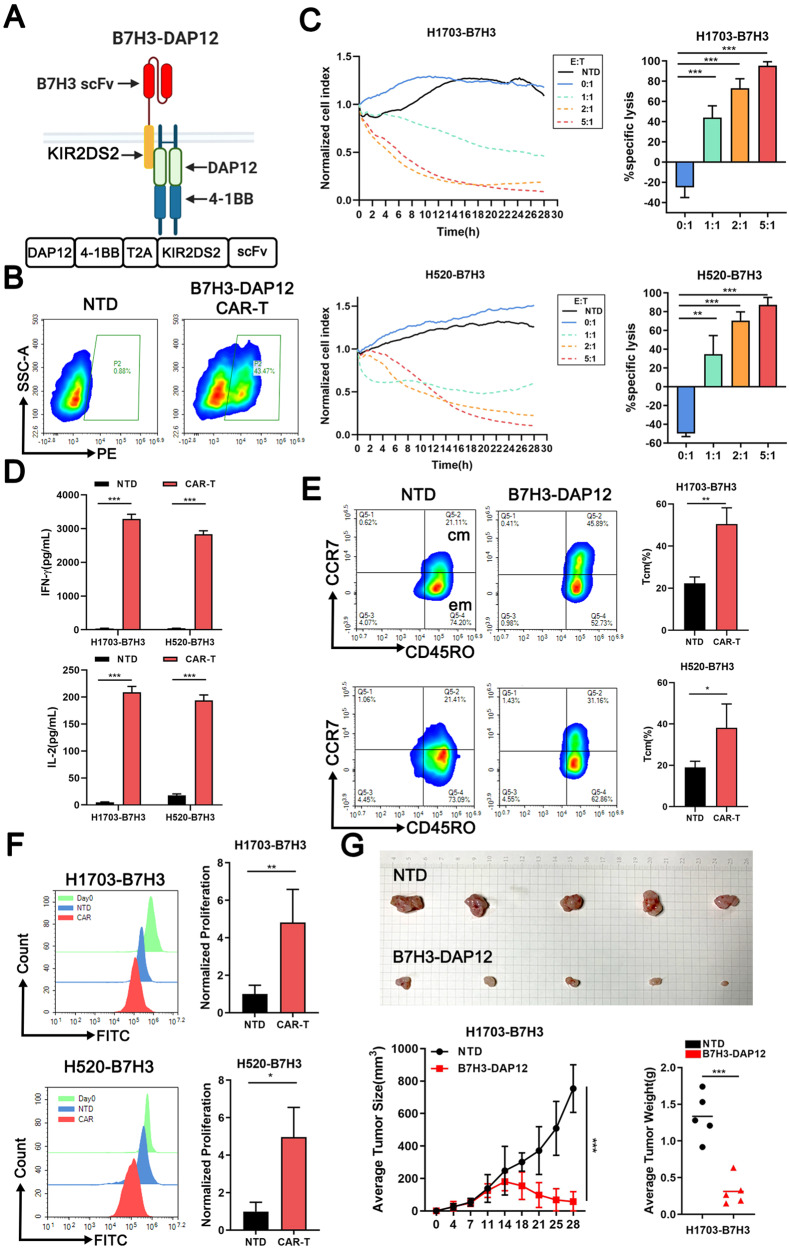
B7H3-DAP12-CAR-T exhibits cell-killing effects on LUSC cells. **A** A schematic representation of the KIR2DS2*/*DAP12-based CAR used in this study is depicted. Figure created using BioRender.com. **B** CAR expression on NTD and B7H3-DAP12-CAR-T shown by flow cytometry. **C** Normalized cell index of B7H3-DAP12-CAR-T (effector cells) co-cultured with LUSC cells (Target cells) (1 × 10^4^) at various effector-to-target (E:T) ratio (0:1, 1:1, 2:1, and 5:1) within 28 h. NTD co-cultured with LUSC cells at an E:T = 5:1 as a negative control (NC). Specific lysis calculated based on the cell index. **D** IL-2 and IFN-γ secreted by B7H3-DAP12-CAR-T or NTD co-cultured with target cells (2 × 10^5^) at E:T = 2:1 for 48 h measured by ELISA. **E** Proliferation of CFSE labeled B7H3-DAP12-CAR-T or NTD co-cultured with target cells at E:T = 1:1 for 3 days shown by flow cytometry. **F** Tcm subsets of B7H3-DAP12-CAR-T or NTD co-cultured with target cells at E:T = 2:1 for 48 h shown by flow cytometry. **G** Subcutaneous tumors constructed by injection of 1703-B7H3 cells (5 × 10^6^ cells/each) on the right side of mice. CAR-T or NTD (1 × 10^7^ cells/each) injected into the tail vein 2 weeks later. Their tumor volume and weight changes were compared with each other. *P < 0.05; **P < 0.01; ***P < 0.001; ns, not significant. Variables are presented as mean ± SD.

A CAR based on Killer Immunoglobulin-Like Receptor 2DS2 (KIR2DS2) was engineered to specifically target B7H3, employing DAP12 instead of CD3z as the activation domain (Fig. 2A). The day of lentivirus infection is defined as day1. After infected for 3 days, the CAR transduction efficiencies approached 50% (Fig. 2B). The introduction of CAR did not affect the proliferation of T cells (Supplementary Fig. S2A). To determine the effects of B7H3-DAP12-CAR-T, B7H3 overexpressed LUSC cell lines were constructed as the target cells, and the efficiency was confirmed by qRT-PCR, flow cytometry, and western blot (Supplementary Fig. S2B–D).

CAR-T and tumor cells were co-cultured at different ratios (E:T = 0:1, 1:1, 2:1, and 5:1). When compared to non-transduced T cells (NTD), B7H3-DAP12-CAR-T effectively eliminated LUSC cells (Fig. 2C). The cytotoxicity was dose-dependent and caused the highest specific lysis at E:T = 5:1 after the 28-hour culturing. The secretion of cytokines, including interferon-gamma (IFN-γ) and interleukin-2 (IL-2), was also determined (Fig. 2D). CAR-T secreted significantly higher levels of IFN-γ and IL-2 than NTD at E:T = 2:1. CAR-T cells were also co-cultured with target cells at E:T = 1:1 from day 9 to day 11, and exhibited more vigorously proliferation when stimulated by target tumor cells than NTD (Fig. 2E).

T cells were divided into four phenotypes based on markers, i.e. naive-like (Tn, CD45RO-CCR7 + ), central memory (Tcm, CD45RO + CCR7 + ), effector memory (Tem, CD45RO + CCR7-), and effector (Teff, CD45RO-CCR7-) T cell subsets. Phenotypic ratios of NTD and CAR-T were measured at day8 (Supplementary Fig. S2E). After co-cultured with target cells for 2 days, CAR-T exhibited a higher ratio of Tcm than NTD (Fig. 2F). Additionally, the apoptosis rates of both CAR-T and NTD were less than 10% (Supplementary Fig. S2F). Finally, the anti-tumor cytotoxicity of B7H3-DAP12-CAR-T cells was evaluated in vivo, and superior control of tumor growth was achieved compared to NTD (Fig. 2G).

### Met deficiency inhibits cytotoxicity of B7H3-DAP12-CAR-T

Previous studies have indicated that depletion of the essential amino acid, Met, impairs T cell functions [15]. To assess the role of amino acid in lung cancer, we analyzed the differential expression of amino acids between patients with lung cancer and healthy individuals [16–18] (Supplementary Fig. S3A). We obtained five pairs of plasma from patients with LUSC and healthy individuals to perform an amino acid profile analysis (Supplementary Table S3). Results revealed a significant decrease of Met in patients with LUSC, which is in agreement with prior studies (Supplementary Fig. S3B). To analyze effects of different amino acid deficiencies, we prepared RPMI-1640 completer medium (CM) with 25% concentrations of specific amino acids (histidine, lysine, threonine, leucine, or methionine). Lactic dehydrogenase (LDH) cytotoxicity assays showed that only co-culturing in Met deficient conditions, cell killing effects of B7H3-DAP12-CAR-T were significantly downregulated (Supplementary Fig. S3C).

**Fig. 3:**
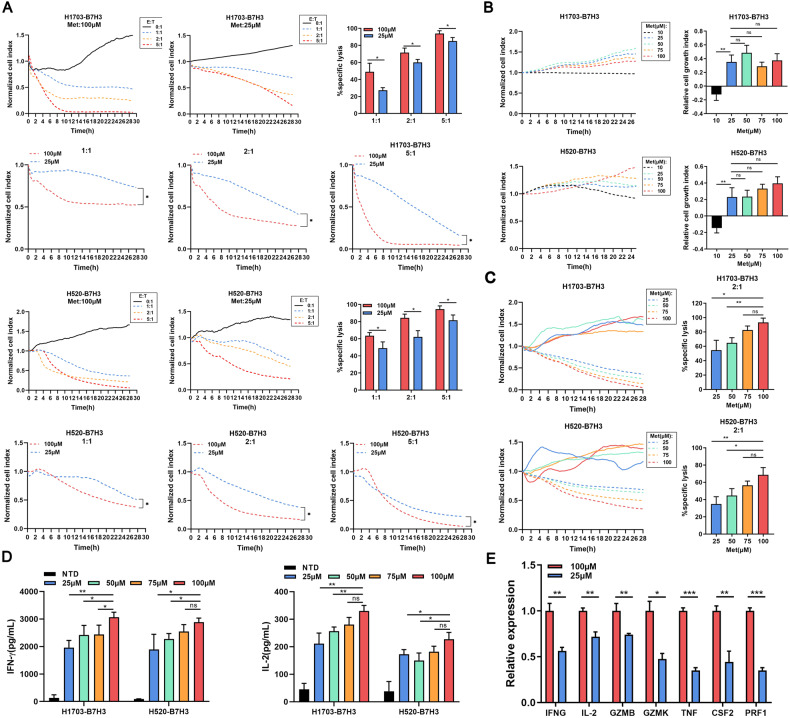
Met deficiency inhibits cytotoxicity of B7H3-DAP12-CAR-T. **A** Normalized cell index of B7H3-DAP12-CAR-T co-cultured with target cells at different ratios (E:T = 0:1, 1:1, 2:1, 5:1) in 25 or 100 μM Met within 28 h. **B** Normalized cell index of LUSC cell lines at 10, 25, 50, 75, or 100 μM Met within 24 h. **C** Normalized cell index of B7H3-DAP12-CAR-T co-cultured with target cells (E:T = 0:1 or 2:1) at 25, 50, 75, or 100 μM Met. **D** IL-2 and IFN-γ secreted by B7H3-DAP12-CAR-T co-cultured with target tumor cells (2 × 10^5^) at E:T = 2:1 for 48 h in 25, 50, 75, or 100 μM Met measured by ELISA. **E** Expression of cytokine-associated genes in CAR-T co-cultured with 1703-B7H3 (E:T = 1:1) at 25 or 100 Met shown by qRT-PCR. *P < 0.05; **P < 0.01; ***P < 0.001; ns, not significant. Variables are presented as mean ± SD.

To examine the impact of Met concentration on cytotoxicity, we co-cultured B7H3-DAP12-CAR-T and target cells at two different Met concentrations (100 and 25 μM) (Fig. 3A). The results showed that low Met concentration significantly reduced the specific lysis of CAR-T at each E:T ratio (1:1, 2:1, and 5:1). Conversely, LUSC cell lines remained resistant to changes of Met concentration, with growth being inhibited only when Met was reduced to 10 μM shown by growth index and CCK-8 (Fig. 3B, Supplementary Fig. S3D). Besides, Met concentrations in CAR-T or 1703-B7H3 were similar in medium with 25 or 100 μM Met. However, intracellular Met of CAR-T co-cultured with 1703-B7H3 in 25 μM Met was significantly lower than that in 100 μM (Supplementary Fig. S3E).

Considering that the cytotoxicity of CAR-T at an E:T ratio of 2:1 was similar to that at 5:1, we further evaluated the effects of different Met concentrations (25, 50, 75, and 100 μM) on CAR-T at 2:1 (Fig. 3C). The cell-killing ability of CAR-T in RPMI-1640 with less than 50 μM Met was significantly inhibited. The secretion of IFN-γ and IL-2 also decreased with descending Met concentrations (Fig. 3D). Furthermore, the expression of cytokine genes including IFNG, IL-2, GZMB, GZMK, and TNF and colony stimulating factor CSF2 and perforin PRF1 was downregulated in CAR-T at 25 μM Met (Fig. 3E). Consistent with above results, CAR-T stimulated by 4-1BB/CD3z showed weaker cytotoxicity and secreted less cytokines in 25 μM Met compared to those in 100 μM (Supplementary Fig. S3F, G).

### Met deficiency attenuates immune effects of B7H3-DAP12-CAR-T

**Fig. 4:**
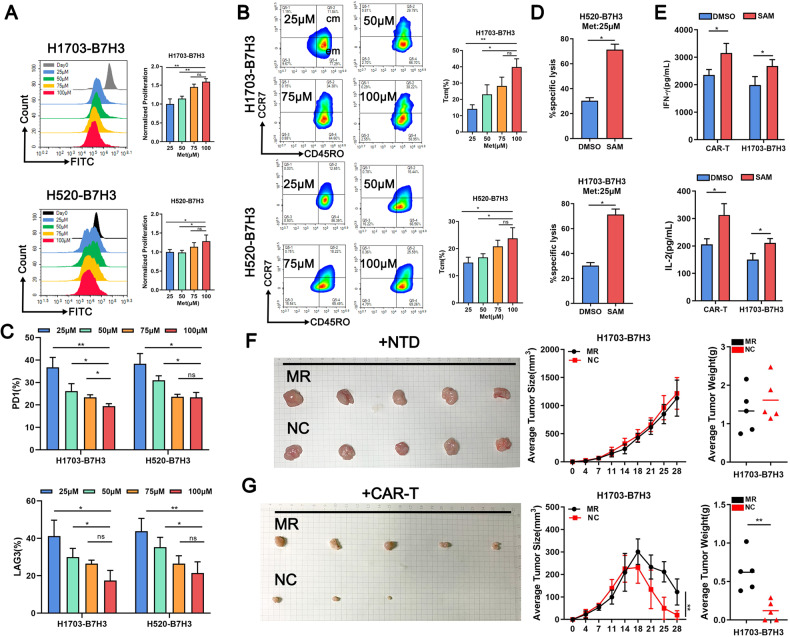
Met deficiency inhibits CAR-T proliferation and differentiation and exacerbates exhaustion. **A** Proliferation of CFSE labeled B7H3-DAP12-CAR-T co-cultured with target cells at E:T = 1:1 at 25, 50, 75, or 100 μM Met for 3 days shown by flow cytometry. **B** Tcm subsets of B7H3-DAP12-CAR-T co-cultured with target cells at E:T = 2:1 for 48 h at 25, 50, 75, or 100 μM Met shown by flow cytometry. **C** Exhaustion markers (PD1 and LAG3) on B7H3-DAP12-CAR-T co-cultured with target cells at 25, 50, 75, or 100 μM Met for 48 h measured by flow cytometry. **D** Subcutaneous tumors constructed by injection of 1703-B7H3 cells (5 × 10^6^ cells/each) on the right side of mice. When the tumor is formed, the Met restriction (MR) feed or negative control (NC) feed was conducted. CAR-T (1 × 10^7^ cells/each) injected into the tail vein 2 weeks later in the treated group. Their tumor volume and weight changes were compared with each other. *P < 0.05; **P < 0.01; ***P < 0.001; ns, not significant. Variables are presented as mean ± SD.

After co-cultured with target LUSC cells for 2 days, the proliferation rate of CAR-T was similar at 25 or 50 μM Met and slightly slower than that at 75 or 100 μM (Fig. 4A). Flow cytometry showed that in day 10, the highest Tcm ratio was observed at 100 μM, whereas lower Tcm ratios were observed at the low Met medium (Fig. 4B). Additionally, the exhaustion degree of CAR-T was determined by PD1 and LAG3 (Fig. 4C, Supplementary Fig. S4A). The most pronounced exhaustion of CAR-T was observed in 25 μM Met. Besides, flow cytometry showed that B7H3 expression on target LUSC cells was unchanged at either 25 or 100 μM Met. However, the positive rate of CAR was higher at 25 μM, which needed more investigation (Supplementary Fig. S4B, C). Ingested Met was converted to S-adenosylmethionine (SAM) to participate in epigenetic methylation [19]. Correspondingly, adding the downstream products SAM could partly restored the tumor killing and cytokines secreting functions of CAR-T at 25 μM Met (Fig. 4D, E). Furthermore, the anti-tumor cytotoxicity of B7H3-DAP12-CAR-T was evaluated in vivo by feeding with or without Met restriction (MR). The intra-tumoral Met concentration in MR group was significantly lower than that in negative control (NC) group (Supplementary Fig. S4D). Although feeding without MR did not affect the growth of subcutaneous tumors, the killing effects of CAR-T were reduced under low Met concentration (Fig. 4F, G).

### Met deficiency reduces m5C modification to downregulate NKG7

**Fig. 5:**
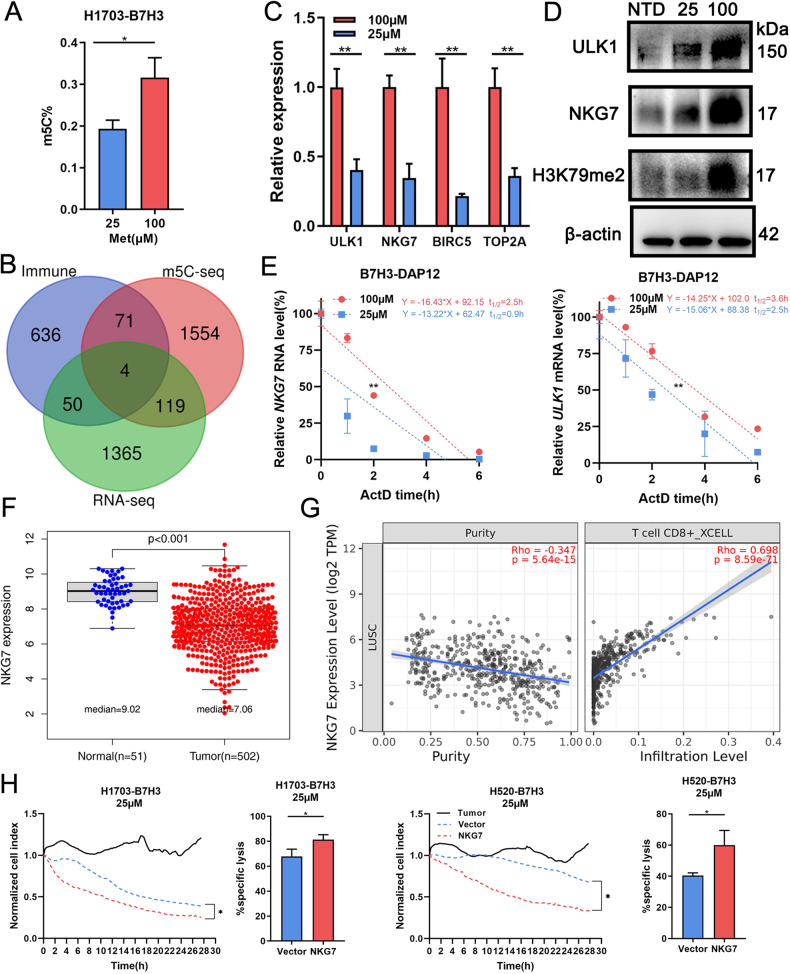
Met deficiency reduces m5C modification to downregulate NKG7. **A** m5C expression in CAR-T co-cultured with target cells (E:T = 1:1) at 25 or 100 μM Met for 2 days detected by ELISA. **B** The Venn diagram consisted of the m5C downregulated gene profile (*n* = 1748), the expression downregulated gene profile (*n* = 1538), and the published immune-related gene profile (*n* = 763). **C**, **D** Gene and protein expression in CAR-T co-cultured with 1703-B7H3 (E:T = 1:1) at 25 or 100 μM Met for 2 days detected by qRT-PCR and WB. **E** After co-cultured with 1703-B7H3 (E:T = 1:1) at 25 or 100 μM Met for 2 days, CAR-T was treated by actinomycin D (2 µg/ml). ULK1 and NKG7 mRNA in CAR-T at indicated time points detected by qRT-PCR. **F** Expression of NKG7 in LUSC (n = 502) and normal samples (*n* = 51) based on TCGA. **G** The relationship between NKG7 and CD8 + T cells (R = 0.698, *P* < 0.001) and tumor purity (R = –0.347, *P* < 0.001) in LUSC analyzed by Timer2.0. **H** Normalized cell index of normal or NKG7 overexpressed B7H3-DAP12-CAR-T co-cultured with target cells (E:T = 2:1) in 25 μM Met within 28 h. *P < 0.05; **P < 0.01; ***P < 0.001; ns, not significant. Variables are presented as mean ± SD.

To determine mechanism of Met deficiency on CAR-T, differently expressed genes in CAR-T co-cultured with 1703-B7H3 at 25 and 100 μM Met were sequenced (Supplementary Table S4). Results revealed that 1,538 genes were significantly downregulated at 25 μM Met compared to those at 100 μM, which were mainly enriched in metabolism and biosynthesis pathways (Supplementary Fig. S5A). Furthermore, ELISA assays demonstrated decreased 5-methylcytosine (m5C) expression in CAR-T at 25 μM Met (Fig. 5A). m5C-RIP sequencing was conducted and 1,748 genes exhibited reduced m5C modification, which were mainly enriched in immune associated pathways including T cell receptor (TCR), natural killer (NK), and helper T cell differentiation (Supplementary Fig. S5B, Supplementary Table S5).

Based on these findings, several genes related to immune were screened for further analysis (Fig. 5B, Supplementary Table S6) [20]. *unc-51-like autophagy activating kinase 1* (*ULK1*) is recently reported to promote T cell survival by mediating autophagy [21]. Besides, *baculoviral IAP repeat containing 5* (*BIRC5*) and *DNA topoisomerase 2 alpha* (*TOP2A*) were classic proliferation markers [22]. *NK cell granule protein-7 (NKG7)* has been reported to play an important role in anti-tumor immunity [23]. These four genes were all downregulated in CAR-T at 25 μM Met (Fig. 5C). Western blotting assay confirmed the decrease of NKG7 and ULK1 protein at 25 μM Met. H3K79me2, which is sensitive to tumor-altered Met metabolism in T cells, was also downregulated (Fig. 5D). Moreover, bisulfite conversion and Sanger sequencing confirmed the loss of m5C modification in NKG7 and ULK1 mRNA (Supplementary Fig. S5C).

Considering that m5C participated in RNA stability, the lack of m5C might result in instability of mRNA [24]. The half-life of NKG7 and ULK1 mRNA in CAR-T at 25 μM Met was significantly shorter than those at 100 μM, suggesting that these mRNAs were degraded more quickly (Fig. 5E).

According to TCGA, NKG7 was lowly expressed in LUSC compared to normal tissues (Fig. 5F). Timer2.0 revealed that in LUSC, NKG7 was positively associated with CD8 + T cells and negatively associated with tumor purity (Fig. 5G). Based on the published single-cell sequencing results, *NKG7* is highly expressed in T cells (Supplementary Fig. S5D) [25]. Moreover, according to single-cell transcriptome database TISCH2 [26], NKG7 is highly expressed in CD8 + T cells and NK cells in NSCLC samples (Supplementary Fig. S5E). KEEG analysis showed that genes whose expression was significantly associated with NKG7 were enriched in NK cell medicated cytotoxicity, cytokine-cytokine receptor interaction, and TCR signaling pathways (Supplementary Fig. S5F). Upregulating NKG7 in CAR-T by lentivirus transduction could promote the cytotoxicity at low Met concentration (Fig. 5H), suggesting that NKG7 is crucial for the anti-tumor function of CAR-T.

### SLC7A5 mainly responsible for Met uptake in LUSC

**Fig. 6:**
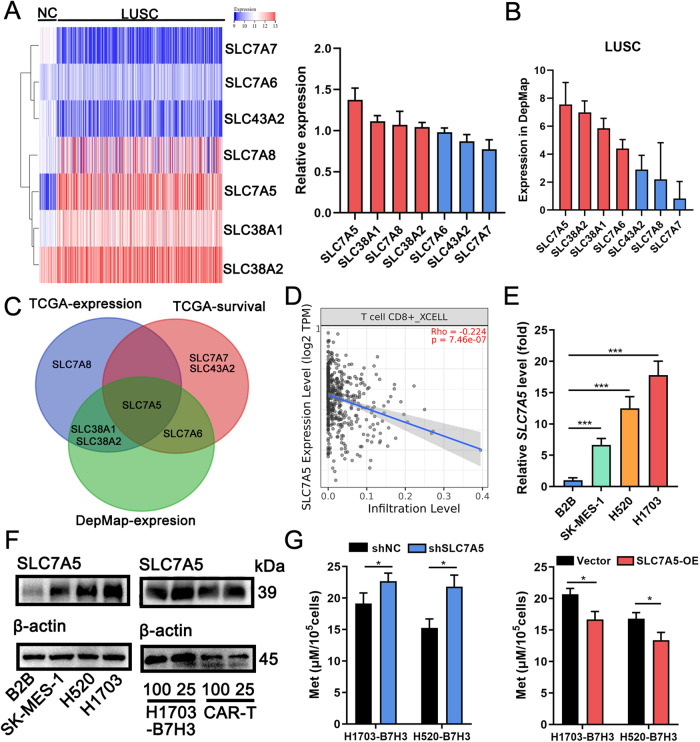
SLC7A5 mainly responsible for Met uptake in LUSC. **A** Expression of classical Met transporters (SLC7A5, SLC7A6, SLC7A7, SLC7A8,SLC38A1, SLC38A2, SLC43A2) in LUSC (*n* = 502) relative to normal adjacent tissues (*n* = 51) based on TCGA shown by the heatmap and bar chart. **B** Expression of SLC7A5 among 26 types of LUSC cell lines based on DepMap. **C** A Venn diagram consisting of SLCs highly expressed in LUSC based on TCGA, SLCs highly expressed in LUSC cell lines based on DepMap, and SLCs negatively associated with the overall survival of LUSC patients based on TCGA. **D** The relationship between SLC7A5 and CD8 + T cells (R = –0.224, *P* < 0.001) in LUSC analyzed by Timer2.0. **E** SLC7A5 expressed in SK-MES-1, H520, H1703, and B2B shown by qRT-PCR. **F** SLC7A5 expressed in SK-MES-1, H520, H1703, and B2B shown by WB (left); SLC7A5 expression in 1703-B7H3 and CAR-T after co-cultured (E:T = 2:1) for 48 h in 25 or 100 μM shown by WB (right). **G** After culturing for 2 days, Met concentration in supernatant of 1 × 10^5^ LUSC cells with SLC7A5 up- or down-regulated detected by ELISA. *P < 0.05; **P < 0.01; ***P < 0.001; ns, not significant. Variables are presented as mean ± SD.

Several solute carriers (SLCs), such as SLC7A5/6/7/8, SLC38A1/2, and SLC43A2, have been found to mediate Met transportation [27]. Analysis of the TCGA database showed that four SLCs were highly expressed in LUSC compared to normal tissues, including SLC7A5, SLC7A8, SLC38A1, and SLC38A2, with SLC7A5 revealed the most remarkable difference (Fig. 6A, Supplementary Fig. S6A). Of all mentioned SLCs, only SLC7A5/6/7 and SLC43A2 revealed a negative association with overall survival, suggesting their high expression contributes to tumor progression (Supplementary Fig. S6B). Based on the DepMap database, SLC7A5/6 and SLC38A1/2 were found to be the top four highly expressed genes among 26 types of LUSC cell lines (Fig. 6B). Therefore, SLC7A5 was selected for further investigation (Fig. 6C). A significant negative correlation between SLC7A5 and CD8 + T cells in LUSC was confirmed by Timer2.0 database (Fig. 6D). qRT-PCR and western blot assays confirmed that SLC7A5 was highly expressed in LUSC cell lines compared to B2B (Fig. 6E-F). To contrast, the differential expression of SLC38A1, SLC38A2, and SLC43A2 in LUSC cell lines and B2B was less significant (Supplementary Fig. S6C). Western blot also showed at 25 μM Met, SLC7A5 was upregulated in both 1703-B7H3 and CAR-T cells (Fig. 6F). To examine its role, the expression of SLC7A5 in LUSC cells was adjusted by shRNA (Supplementary Fig. S6D). After 2 days of culturing, the Met concentration was higher in the supernatant of SLC7A5 downregulated cells, and lower in the supernatant of SLC7A5 upregulated cells (Fig. 6G). On the other side, Leu concentration remained similar in the supernatant of SLC7A5 up- or down-regulated 1703-B7H3 cells (Supplementary Fig. S6E). These results suggested that SLC7A5 was mainly responsible for the Met uptake in LUSC. In addition, SLC43A2 knockdown also increased Met concentration in supernatant while SLC38A2 knockdown had no effects (Supplementary Fig. S6F). However, at 25 μM Met, SLC7A5 was upregulated in 1703-B7H3 and CAR-T but SLC43A2 was downregulated (Supplementary Fig. S6G). The role of SLC43A2 in Met uptake of LUSC deserves further investigation.

### SLC7A5 knockdown in target cells rescues functions of B7H3-DAP12-CAR-T at low Met concentration

**Fig. 7:**
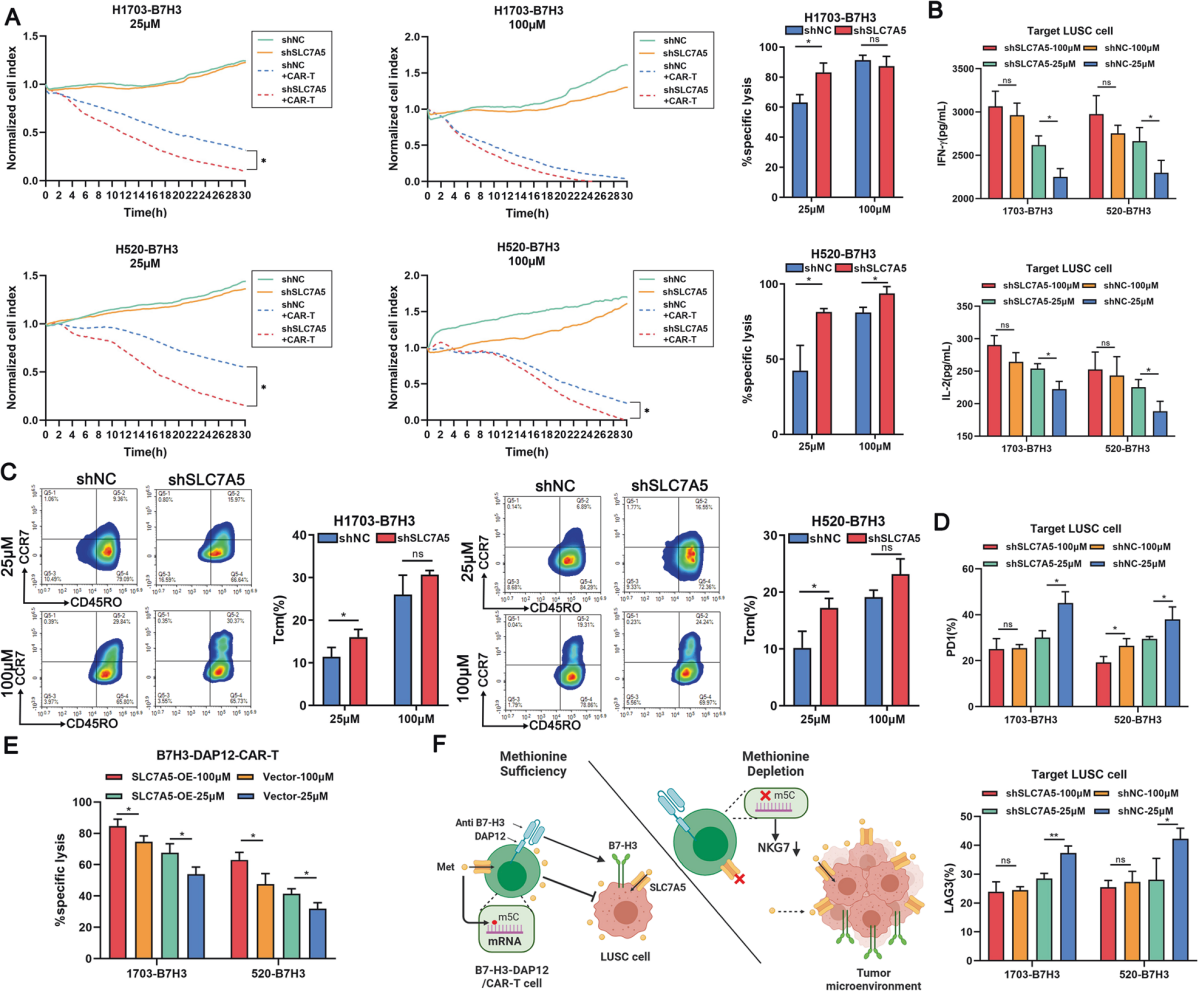
SLC7A5 knockdown in target cells rescues functions of B7H3-DAP12-CAR-T at low Met concentration. **A** Normalized cell index of B7H3-DAP12-CAR-T co-cultured with normal or SLC7A5 downregulated target cells (E:T = 0:1 and 2:1) in 25 or 100 μM Met within 30 h. **B** IL-2 and IFN-γ secreted by B7H3-DAP12-CAR-T co-cultured with normal or SLC7A5 downregulated target cells (2 × 10^5^) at E:T = 2:1 for 48 h in 25 or 100 μM Met measured by ELISA. **C** Tcm subsets of B7H3-DAP12-CAR-T co-cultured with normal or SLC7A5 downregulated target cells at E:T = 2:1 for 48 h at 25 or 100 μM shown by flow cytometry. **D** Exhaustion markers (PD1 and LAG3) on B7H3-DAP12-CAR-T co-cultured with normal or SLC7A5 downregulated target cells at 25 or 100 μM for 48 h measured by flow cytometry. **E** Cytotoxicity of normal or SLC7A5 upregulated B7H3-DAP12-CAR-T co-cultured with 1 × 10^4^ LUSC cells (E:T = 2:1) for 24 h at 25 or 100 µM Met shown by LDH cytotoxicity assay. **F** In a Met-sufficient environment, CAR-T cells equipped with B7H3 scFv can recognize B7H3-positive LUSC, activate the DAP12 stimulating domain, and exert killing function. In a Met-deficient environment, LUSC cells with high expression of SLC7A5 would compete for Met uptake, resulting in reduced Met absorption by CAR-T cells and a subsequent decline in the m5C modification within CAR-T cells. The reduction in m5C would compromise the stability of NKG7 mRNA, leading to the downregulation of NKG7 expression and subsequently inhibiting the cytotoxicity of CAR-T cells. *P < 0.05; **P < 0.01; ***P < 0.001; ns, not significant. Variables are presented as mean ± SD.

We postulated that reducing Met uptake in LUSC by downregulating SLC7A5 may improve CAR-T function. SLC7A5 knockdown had slight negative impacts on the growth of LUSC cell lines within 30 h (Fig.7A). CAR-T co-culturing with SLC7A5 knockdown LUSC cells showed increased specific lysis, which was more pronounced at 25 μM Met. To contrast, the difference between CAR-T co-cultured with shNC or shSLC7A5 LUSC cells at 100 μM was limited, potentially due to the sufficiency of Met in the complete medium (Fig. 7A). The production of IFN-γ and IL-2 was also significantly upregulated at 25 μM but not at 100 μM (Fig. 7B). Nevertheless, the IFN-γ and IL-2 secretion levels at 25 μM remained lower than those at 100 μM. Co-culturing with SLC7A5 knockdown LUSC cells also resulted in a significantly increased proportion of Tcm phenotype at 25 μM Met (Fig. 7C). Only at 25 μM Met, CAR-T co-culturing with shSLC7A5 LUSC cells exhibited lower PD1 and LAG3 than those with shNC LUSC cells (Fig. 7D, Supplementary Fig. S7A). However, SLC7A5 knockdown had little effects on the proliferation of CAR-T at neither 25 μM nor 100 μM (Supplementary Fig. S7B). On the contrary, co-culturing with SLC7A5 overexpressed H1703-B7H3 cells inhibited the cytotoxicity, cytokines expression, and Tcm phenotype of CAR-T cells especially at 100 μM (Supplementary Fig. S7C–F). In addition, upregulated SLC7A5 on CAR-T could promote their cell killing effects and cytokines secretion at either 25 or 100 μM Met (Fig. 7E).

## Discussion

Our previous study has demonstrated the advantages of DAP12 based CAR over traditional second-generation CAR [13]. DAP12 is a short 12kDa transmembrane protein expressed on the surface of multiple immune cells [28], and contains an immunoreceptor tyrosine-based inhibitory motif that prevents CAR tonic signaling [29]. CAR-NK cells with DAP12 also showed promising effects on patients with metastatic colorectal cancer [30]. Our Phase I clinical trial presented that CAR-T activated by DAP12 targeting CD19 was safe and efficient [13]. In light of these findings, we employed DAP12 to provide the stimulation signaling. In this study, B7H3 was identified highly expressed in LUSC. DAP12-CAR-T targeting B7H3 exhibited marked specific lysis against LUSC cells both in vitro and in vivo. Additionally, B7H3-DAP12-CAR-T secreted more cytokines and showed a quicker rate of proliferation and a higher ratio of Tcm compared to NTD, but with similar apoptosis rates.

By analyzing the amino acid profiles of patients with lung cancer, Met was observed to be one of the most disturbed amino acids [16–18]. Our study confirmed this discrepancy by analyzing the plasma of patients with LUSC and healthy individuals. When co-cultured in media with different amino acid deficiencies, the killing function of CAR-T cells showed the most pronounced decrease in the low Met environment. Met is known to play an important role in T cell function, and its restriction in the TME has been linked to loss of H3K79me2, which can impair the anti-tumor immune response of T cells [15]. Our results revealed that Met deficiency had a limited effect on the growth of LUSC cells, but a detrimental effect on CAR-T. At 25 μM Met, the content of Met was significantly reduced in co-cultured CAR-T, but not in tumor cells. Following the decrease of Met, the cytotoxicity, cytokine secretion, and phenotype differentiation of CAR-T was inhibited, and exhaustion markers including PD-1 and LAG3 were increased. CAR-T cell proliferation also decreased significantly, but to a lesser extent. However, in a low Met environment, the positivity of CAR on T cells was rather higher. Further studies about this paradox are required. In vivo experiments showed that Met restriction did not affect tumor growth, but the efficacy of B7H3-DAP12-CAR-T was inhibited due to the Met-restricted feedstuff. In our previous study, we observed that the BBζ CAR expression was impaired in the TME that also lacked Met. In contrast, the KIR/DAP12 CAR expression was preserved [31]. The inhibitory effects of low Met on cytotoxicity and cytokine secretion were also demonstrated in CD3z-CAR-T. These results highlight the significance of Met in the tumor clearance capacity of CAR-T.

Notably, Met serves as the donor for cellular epigenetic methylation [32]. In the cell, Met is converted to SAM to provide methylation sources [33]. Supplementing SAM in the medium with low Met could partly restore the cytotoxicity of CAR-T. Besides, Met determines the amount of m5C in mRNA [32], which plays a significant role in mRNA modification such as nuclear export, maturation, and stability [34]. The distribution and variation of m5C affected biological activities and cytokine expression of T lymphocytes [35, 36]. To explore the mechanism underlying the immunosuppression of Met decrease, co-cultured CAR-T at 25 or 100 μM Met was checked by RNA-seq and m5C-RIP-seq. At 25 μM Met, 1,748 genes exhibited decreased m5C modification, and 1,538 genes were significantly downregulated in CAR-T. Among downregulated genes with reduced m5C modification, NKG7 is reported to play a key role in T cell immunity. Patients resistant to the anti-PD1 therapy had low levels of NKG7 [23], which is highly expressed by immune cell subsets including CD4 + T cells, CD8+ cells, and NK cells and necessary for the cytotoxic function [37]. Low Met environment resulted in the reduction of m5C and the instability of NKG7 mRNA, which may contribute to the repression of the tumor-killing capacity of CAR-T. Upregulation of NKG7 could promote the cytotoxicity of CAR-T at the low Met concentration, suggesting that the expression of NKG7 is critical for the efficacy of CAR-T in TME.

The mechanism underlying the cellular uptake of Met has been investigated [27]. It has been reported that SLC43A2 is mainly responsible for the Met uptake of melanoma cells [15]. However, SLC43A2 was found to be lowly expressed in LUSC compared to normal tissues and downregulated in the low Met concentration. Several other SLCs including SLC7A5/6/7/8 and SLC38A1/2 were also evaluated for their involvement in Met transport [27]. Particularly, SLC7A5 was highly expressed in LUSC and predicted poor prognosis in LUSC patients. SLC7A5 was increased in CAR-T and LUSC cells at the low Met concentration. Thus, SLC7A5 may play a key role in the Met uptake of cells. SLC7A5 knockdown decreased Met consumption of LUSC cells. At low Met concentrations, B7H3-DAP12-CAR-T co-cultured with SLC7A5 knockdown LUSC cells showed greater cell-killing effects and lower exhaustion markers. On the other hand, at 100 μM Met, B7H3-DAP12-CAR-T co-cultured with SLC7A5 overexpressed LUSC cells showed suppressed immune function. However, there was no significant change in CAR-T proliferation, suggesting that the effect of Met on proliferation was limited. In addition, overexpressing SLC7A5 in CAR-T helped restore killing functions. Precise modulation of Met uptake capacity of CAR-T cells has the potential to facilitate their clinical application.

## Conclusion

Our study demonstrated that B7H3-DAP12-CAR-T could efficiently eliminate LUSC cells. However, LUSC cells outcompeted CAR-T for Met by SLC7A5. Met deficiency induced the downregulation of NKG7, leading to the inhibition of cytotoxicity (Fig. 7F). Improved Met uptake of CAR-T may be expected to enhance killing function and avoid T cell exhaustion, thereby improving individualized treatment outcomes.

## Materials and Methods

### Clinical specimens

Ten cases each of normal lung tissues, lung squamous cancer tissues (LUSC), lung adenocarcinoma tissues and small cell lung cancer tissues were collected from the Department of Pathology, Jiangsu Provincial People’s Hospital from February 2018 to February 2020. LUSC tissue microarray (TMA) was purchased from Wuhan servicebio technology (SLC-1901), containing 48 pairs of LUSC tissue specimens and normal tissue specimens. Samples were collected ranging from August 2013 to July 2015. Immunohistochemical Staining (IHC) was performed by anti-B7H3 (Lito, China, LTO5825) antibody combined with automated quantitative analysis. The dilution of primary antibodies was 1:100. Slides were examined independently by ImageJ. The protocol was assessed by the Ethics Committee of the People’s Hospital of Jiangsu Province (No. 2021-SRFA-342).

### Construction of CAR

The DAP12-4-1BB-T2A-B7H3 scFv-KIR2DS2-expressing lentiviral vectors were constructed following the previous protocol [38]. DAP12 cDNA was cloned from human peripheral blood mononuclear cells (PBMC) using the following primers: 5_-*TCTAGA*ATGGGGGGACTTGAAC-_3 (XbaI/ is italic), 5_-*GTCGAC*TTTGTAATACGGCCTC_-3 (SalI/ is italic) [31]. The resulting PCR product was cloned in-frame 3′ to a dsRed-Thoseasigna virus 2A (T2A) fusion sequence downstream of the EF-1α promoter in the lentiviral vector. The scFv coding sequence of B7H3 is referenced to the cloned TE9 sequence in the published literature [39]. Synthesized scFv was bound to PCR amplification products of KIR2DS2: 5_-*GCTAGCG*GTGGCGGAGGTTCTGGAGGTGGGGGTTCCTCACCCACTGAACCAAGC _-3 (NheI/ is italic), and 5′_- *GTCGAC*TTATGCGTATGACACC_-3 (SalI/ is italic). The obtained chimeric B7H3 scFv-KIR2DS2 was combined with DAP12-4-1BB-T2A-dsRed to generate the pELPS DAP12-4-1BB-T2A-B7H3 scFv-KIR2DS2 lentiviral plasmid. In simple terms, 1 × 10^7^ HEK293T cells were transfected with pRSV-Rev, pMDLg-pRRE, and pVSV-G and the plasmid pELPS containing the CAR by PEI (Polysciences, USA). 48 h later, supernatant was collected and concentrated by ultracentrifugation. Viral titer was determined by serial dilution and infection of HEK293T cells. The full sequence of B7H3-DAP12-CAR-T could be found in the patent CN115368470A.

Besides, B7H3 scFv, 4-1BB, and CD3z were expressed on the lentiviral vector GV400, which is provided by Shanghai Genechem Co.,Ltd., to generate the second generation of CAR targeting B7H3.

### Expansion of CAR-T

CD3 + T cells of three different sources were purchased from Leide Biosciences Co., Ltd. T cells were cultured in X-vivo medium (LONZA, USA, BE02-060F) supplemented with 5% fetal bovine serum (FBS) and 300 U/ml interleukin 2 (IL-2). Anti-CD3/CD28 Dynabeads (Thermo, USA, 11132D) were utilized for stimulation (25 μl/1 × 10^6^cells). The day T cells infected with lentivirus was recorded as day 1. T cells were exposed to CAR lentivirus from day 1 to day 3. Dynabeads were removed at day 6. Number and size of CAR-T were measured by the Multisizer 4e Coulter Counter (Beckman Coulter, California, USA).

### Flow cytometry analysis

For positive rate analysis, CAR-T was incubated with biotinylated goat antimouse F(ab)2 (Jackson ImmunoResearch, USA, 109-066-006) for 30 min, followed by streptavidin-PE (BD Biosciences, 554061). Flow cytometry was performed by CantoII (BD Biosciences) and analyzed by NovoExpress (Agilent, USA). B7H3 PE-conjugated antibody (Biotechne, FAB1027P) was used for the detection of B7H3 on the cell membrane. Anti-human CD3-APC (BD Biosciences, 555342), anti-CCR7-BV421 (BD Biosciences, 740052) and anti-CD45RO-APC (ThermoFisher, 17-0457-42) antibodies were used for evaluation of the CAR-T differentiation. APC-conjugated PD1 and LAG3 antibodies (ThermoFisher, 17-9981-82, 17-2239-42) were used to reflect the exhaustion. The apoptosis of CAR-T was detected after co-cultured with target cells at E:T = 1:1 for 3 days by Annexin V-FITC and PI (Vazyme, China, A211-01).

### Cell proliferation

To detect the proliferation of suspension cell, cells were stained with Carboxyfluorescein diacetate, succinimidyl ester (CFSE) according to the manufactures (Beyotime, China, C0051). In brief, non-transduced T cells (NTD) or CAR-T were washed by PBS and stained with 5 mM CFSE for 10 min at 37°C. Subsequently, CAR-T was washed twice by RPMI-1640 and incubated with target cells at effector: target (E:T) ratio=1:1 for 3 days. The proliferation rate was analyzed by flow cytometry.

Proliferation of adherent cells was assayed by CCK8 (Beyotime, China, C0037). Cells were cultured in the 96-well plate with 200 µl medium (5 × 10^3^ cells/well). After the adherence, CCK8 was added at a ratio of 1:10. Finally, the absorbance (Optical Density, OD) was measured to reflect the cell growth.

### Cell culture and lentivirus infection

LUSC cell lines (H1703, H520, and SK-MES-1) and the pulmonary epithelial cell BEAS-2B (B2B) were purchased from China Center Type Culture Collection (CCTCC, Shanghai) and cultured in RPMI-1640 (GIBCO, USA) supplemented with 10% FBS, 100 U/ml penicillin and 100 mg/ml streptomycin (Invitrogen, USA) at 37°C and 5% CO2. Authentication by STR profiling and confirmation of mycoplasma contamination-free status were performed prior to the experiments. The amino acid content of RPMI-1640 was as follows: Glycine 133 µM, Arginine 1150 µM, Asparagine 378 µM, Aspartic acid 150 µM, Cystine 207 µM, Glutamic acid 136 µM, Glutamine 2055 µM, Histidine 96 µM, Proline 174 µM, Isoleucine 381 µM, Leucine 381 µM, Lysine 281 µM, Methionine 100 µM, Phenylalanine 90 µM, serine 285 µM, threonine 168 µM, tryptophan 24.5 µM, tyrosine 111 µM, valine 171 µM.

The overexpression vector and shRNA lentivirus were constructed by ShangHai GenePharma Company. After infecting by lentivirus for 3 days and screened by puromycin (1 μg/μl) for 3 days, gene expression was validated by qRT-PCR or western blotting.

### Cytotoxicity assays

Target LUSC cells were cultured in the E-Plate (1 × 10^4^ cells/well) (Agilent, USA, 5469830001). After adherence, NTD or CAR-T were added to the E-Plate at different E:T ratios. The normalized cell index (CI) was automatically recorded by xCELLigence Real-Time Cell Analyzer (RTCA) (Agilent, USA) every 15 min and monitored for approximately 30 h.

$$\% {\rm{Specific\; lysis}}=({\rm{CI}}({\rm{No\; Effector}})-{\rm{CI}}({\rm{Effector}}))/{\rm{CI}}({\rm{Effector}})\times 100 \%$$

Lactate dehydrogenase (LDH) release assay was also applied for the determination of the cytotoxicity and conducted followed the instruction (Beyotime, China, C0016). 1 × 10^4^ target cells were cultured in 96-well plates and washed by PBS. After the adherence, CAR-T was added in the ratio of E:T = 2:1 and co-cultured for 24 h. Before the detection, 20 µl LDH release reagent was added to the control wells as the positive control. The supernatant of each well was centrifuged and mixed with 60 µl LDH working solution, and incubated at room temperature for 30 min away from light. The absorbance was measured at 490 nm.

$$\% {\rm{Specific\; lysis}}=({\rm{treated\; samples}}-{\rm{negative\; control}})/({\rm{positive\; control}}-{\rm{negative\; control}})\times 100 \%$$

### Cytokine production

NTD and CAR-T were co-cultured with 2 × 10^5^ target LUSC cells at E:T = 2:1. After 24 h, supernatants were collected to measure IL-2 and IFN-γ by corresponding enzyme-linked immunosorbent assays (ELISAs) (MAISHA, China, MS10100-B, MS10004-B) according to the manufacturer’s instructions.

### Quantitative real-time PCR (qRT-PCR) assay

Total RNAs were extracted by RNAeasy Plus Animal RNA Isolation Kit (Beyotime, China, R0026) following the instruction. For qRT-PCR, 1 μg of total RNA was reverse-transcribed to cDNA by the HiScript III RT SuperMix for qPCR (Vazyme, China, R323-01) and amplified by ChamQTM SYBR qPCR Master Mix (Vazyme, China, Q341-02). Expression of β-actin was settled as an internal control. All primers (Supplementary Table S1) were provided by Geneseed Biotechnology Company, China.

### Western blotting

Protein was extracted by RIPA with PMSF and their concentration was measured by BCA protein assay kit (Beyotime, China). Proteins were separated by 12% SDS-polyacrylamide gel electrophoresis (SDS-PAGE), transferred to 0.22 µm polyvinylidene fluoride membranes and incubated with blocking buffer or primary antibodies, and following secondary antibodies. Blots were exposed by enhanced chemiluminescence reagent (NCM Biotech, China, P10300). Antibodies were listed: anti-B7H3 (Lito, China, LTO5825), anti-SLC7A5 (Proteintech, China, 28670-1-AP), anti-NKG7 (CST, USA, #84835), anti-ULK1 (Beyotime, China, AF8307), anti-H3K79me2 (Proteintech, China, 39923), and anti-β-Actin (CST, USA, #3700).

### Immunofluorescence (IF)

The IF assay was implemented by the Immunol Fluorescence Staining Kit following the instructions (Beyotime, China). Cells were fixed by 4% paraformaldehyde and permeabilized by 0.5 % Triton X-100. Subsequently, cells were blocked for 1 h and incubated with primary antibodies overnight and with fluorescent secondary antibodies for 2 h in the dark. Pictures were photographed by the confocal laser scanning microscope at a 40 × magnification.

### RNA decay measurements

CAR-T co-cultured with 2 × 10^5^ target cells at E:T = 2:1 in 6-wells plates was collected after 48 h. Transcription was inhibited by actinomycin D (2 µg/ml, MedChemExpress, USA). RNA was regularly extracted by RNAeasy Plus Animal RNA Isolation Kit at 0, 1, 2, 4, and 6 h. Half-life was calculated by drawing linear relationships with time as X-axis and amount of RNA as Y-axis.

### RNA bisulfite conversion

Extracted RNA was converted by EZ RNA Methylation kit (Zymo Research, USA, R5001). In brief, 1 μg RNA was mixed with RNA Conversion Reagent and placed in 70°C for 5 min and 54°C for 45 min. Converted RNA was desulphonated for 30 min. The recovered RNA was amplified by qRT-PCR and the conversion of C to T was sequenced by Sangon Biotech (Shanghai, China).

### RNA seq and m5C-RIP-seq

CAR-T was co-cultured with 1 × 10^6^ target cells at E: T = 1:1 for 48 h. CD3 + CAR-T cells were collected by flow cytometry. RNA was extracted and the concentration and purity were measured. OD260/OD280 within 1.8–2.1 was considered qualified. RNA sequencing was conducted by The Beijing Genomics Institute. PolyA RNA was purified from total RNA using NEBNext Poly(A) mRNA Magnetic Isolation Module. Library was constructed by VAHTS Stranded mRNA-seq Library Prep Kit (Vazyme, China). Each group was sequenced in triplicate. The |log2(FoldChange)| ≥ 0.58 and *P*-value ≤ 0.05 genes were selected to be considered as the significant difference. The sequencing data have been deposited to the Sequence Read Archive (SRA) (PRJNA953945).

M5C sequencing was conducted by Shanghai Genechem Co.,Ltd. M5C-IP and library preparation were performed according to the published protocol [40]. Agilent 2200 system with HS RNA Reagent was used for RNA quality control. Purified RIP RNAs were reverse transcribed into cDNA sequencing library. Libraries were sequenced via Nova platform (Illumina). The sequencing data have been deposited to the SRA (PRJNA 957745).

### Detection of m5C modification

The m5C modification in CAR-T was detected by 5-mC RNA Methylation ELISA Easy Kit (Fluorometric)(Epigentek, USA, P-9009-48). RNA was extracted from CAR-T co-cultured with LUSC cells at E:T = 1:1 at 25 or 100 μM Met for 48 h. After incubating in assay wells at 37°C for 90 min, RNA was mixed with prepared m5C complex solution including m5C antibody. The plate was washed 3 times after removing the diluent and incubated for 50 min with 50 µl of detection solution. After washing, 50 µl of fluorescence developer solution was added and the luminescence was detected at 530 nm excitation light.

### Detection of amino acid in peripheral blood

The amino acid concentration of peripheral blood was measured by Standard Group (Qingdao, China). 5 ml peripheral blood samples were extracted from morning fasting patients with LUSC and healthy people. The serum was processed by centrifugation. The metabolite characterization and principal component were analyzed by Liquid Chromatography Mass Spectrometry (LCMS). The protocol was reviewed by the Ethics Committee of Jiangsu Provincial People’s Hospital (No. 2021-SRFA-342). Metabolites with |log2(FoldChange)| ≥ 0.58 and *P*-value ≤ 0.05 were selected as significant differences.

### Detection of Met and Leu in supernatant and cell

After cultured for 48 h, cells were harvested for counting and the supernatant was centrifuged for measurement. Met or Leu in supernatant and cell was detected by ELISA (Jiangsu Meimian, China, 25719/25739). Purified Met or Leu antibody was coated in microtiter plate wells. Met in samples could combine with antibodies with HRP to form the complex. After completely washing, substrate solution was added to catalyze HRP. The reaction is terminated by the addition of a sulfuric acid solution and the color change is measured spectrophotometrically at a wavelength of 450 nm.

### In vivo tumor experiments

Four-week-old female NCG mice (GemPharmatech, China, T001475) were fed with diets with or without Met (Trophic, China, TP01A0401). Met-restricted (MR) feeds contained 0.12% Met, normal cystine, and the equivalent nitrogen content and capacity. Normal control (NC) feeds had 0.46% Met. Tumor cell mass (5 × 10^6^ cells) in 100 µl PBS were subcutaneously injected into the right side of each mouse. After the numbering, the generated random sequence was matched to the animal number, and the animals were grouped according to the random sequence. Tumor volumes were regularly calculated by measuring the longitudinal diameter and latitudinal diameter. After 2 weeks, mice with visible tumor burden were randomly separated into NTD or CAR-T treatment group (*n* = 5 per group). 1 × 10^7^ NTD or CAR-T cells were injected intravenously via the tail vein. The tumor was also measured every 3–5 days in the following two weeks until the mice were euthanized. Animal experiments with a sample size of 5 per group were determined based on the probability level of 0.05 and power of 0.80. Animal experiments were not conducted in a double-blind manner. The protocol was approved by the Committee on the Ethics of Animal Experiments of the Nanjing Medical University (IACUC-2201042).

### Bioinformatic analysis

Gene expression data of LUSC cell lines was downloaded from DepMap (https://depmap.org/portal/depmap/) [41]. EstimateScores of samples were calculated by ESTIMATE algorithms [42]. Differential expression analysis and survival analysis of LUSC was conducted based on the database of The Cancer Genome Atlas (TCGA) (https://cancergenome.nih. gov/) and GSE157011(n = 484) [43]. Pan-cancer analysis was conducted based on GEPIA (http://gepia.cancer-pku.cn/index.html) [44]. Immune associated gene profiles were downloaded from ImmPort [20]. Single-cell transcriptome data was analyzed by TISCH2 [26].

### Statistical analysis

All in vitro experiments were performed in triplicate and repeated three times. The sample size was determined based on a power analysis, considering the desired power level and alpha level. Experimental data were illustrated by GraphPad Prism 8.0 software. The statistical significance between different groups was computed by unpaired Student’s t-test or two-way analysis of variance (ANOVA). Difference and correlation analyses were performed using R software (version 4.1.1, www.r-project.org). *P* < 0.05 was considered statistically significant. The results presented are representative of one experiment out of three independent trials, and the data are expressed as means ± standard deviation (SD).*P < 0.05; **P < 0.01; ***P < 0.001. ns, not significant.

### Supplementary information

aj-checklist

Supplementary material

Supplementary Table S1

Supplementary Table S2

Supplementary Table S3

Supplementary Table S4

Supplementary Table S5

Supplementary Table S6

Western Blots

## Data Availability

Data are available on reasonable request.

## Code availability

The R code used to analyze the data in this paper is available upon reasonable request. Interested investigators may contact the corresponding author for access to the code.
