# Supplementary material for: Effects of methionine deficiency on B7H3-DAP12-CAR-T cells in the treatment of lung squamous cell carcinoma
Source: Cell Death Dis. 2024 Jan 5;15(1):12. doi: 10.1038/s41419-023-06376-w (PMC10770166; doi:10.1038/s41419-023-06376-w)
Supplement: Supplementary file 2 — Supplementary material [file 41419_2023_6376_MOESM2_ESM.docx]

Supplementary Figures


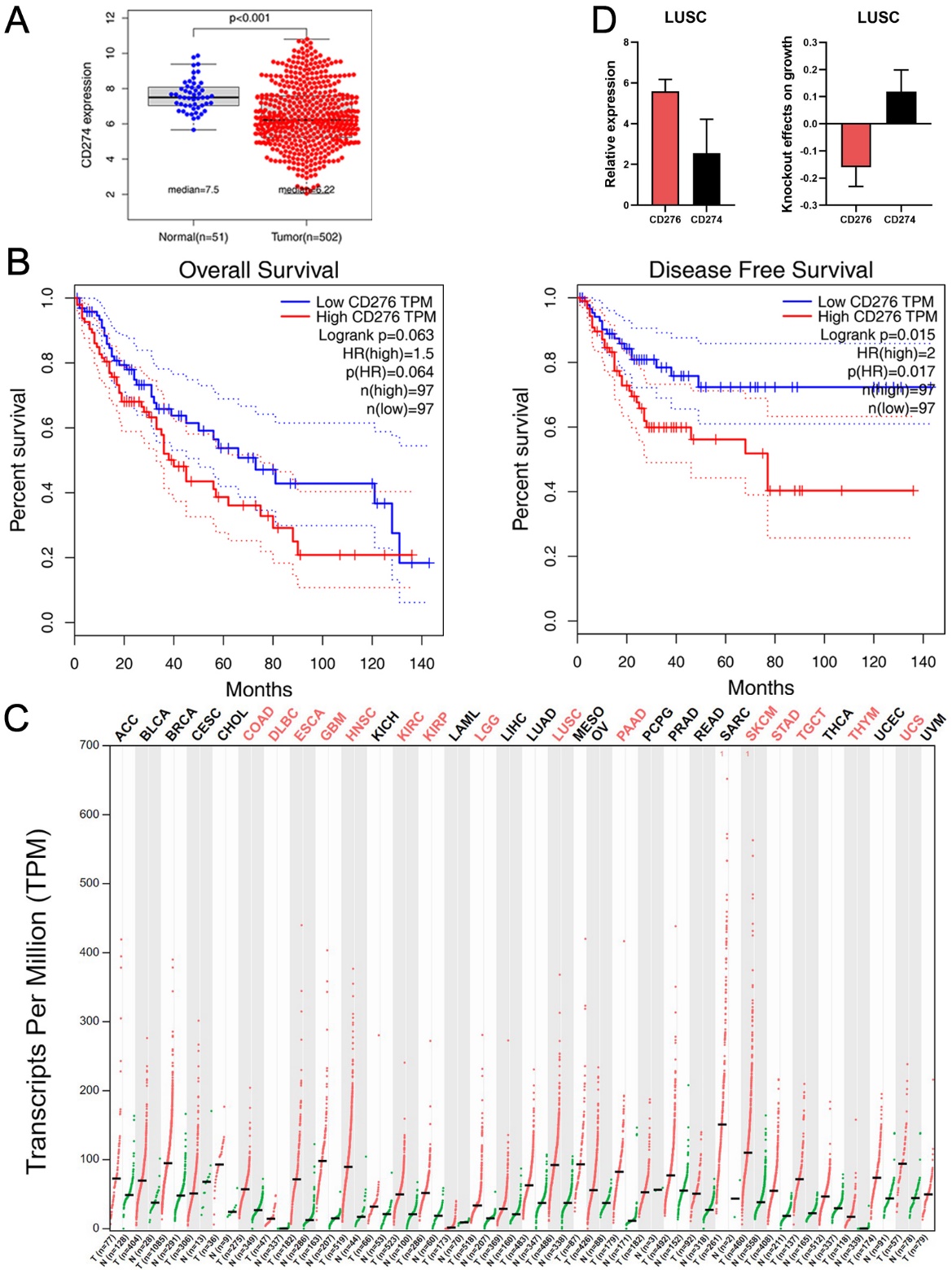


**SFig.1** **A.** Expression of CD274 in LUSC (n=502) and normal samples (n=51) based on TCGA. **B.** Difference in overall and disease-free survival (*P*=0.063, *P* =0.015, respectively) between the top 20% and bottom 20% of LUSC patients (n=97) with CD276 expression analyzed by GEPIA. **C.** CD276 expression in 33 cancers and normal tissues analyzed by GEPIA, and cancer types with significant differences in expression marked by red. **D.** CD276 and CD274 expression in 28 LUSC cell lines downloaded from DepMAp(left). Effects of CD276 or CD274 knockout on the growth of 22 LUSC cell lines analyzed by DepMap (right). * *P* < 0.05; ** *P* < 0.01; *** *P* < 0.001. ns, not significant. Variables are presented as mean ± SD.


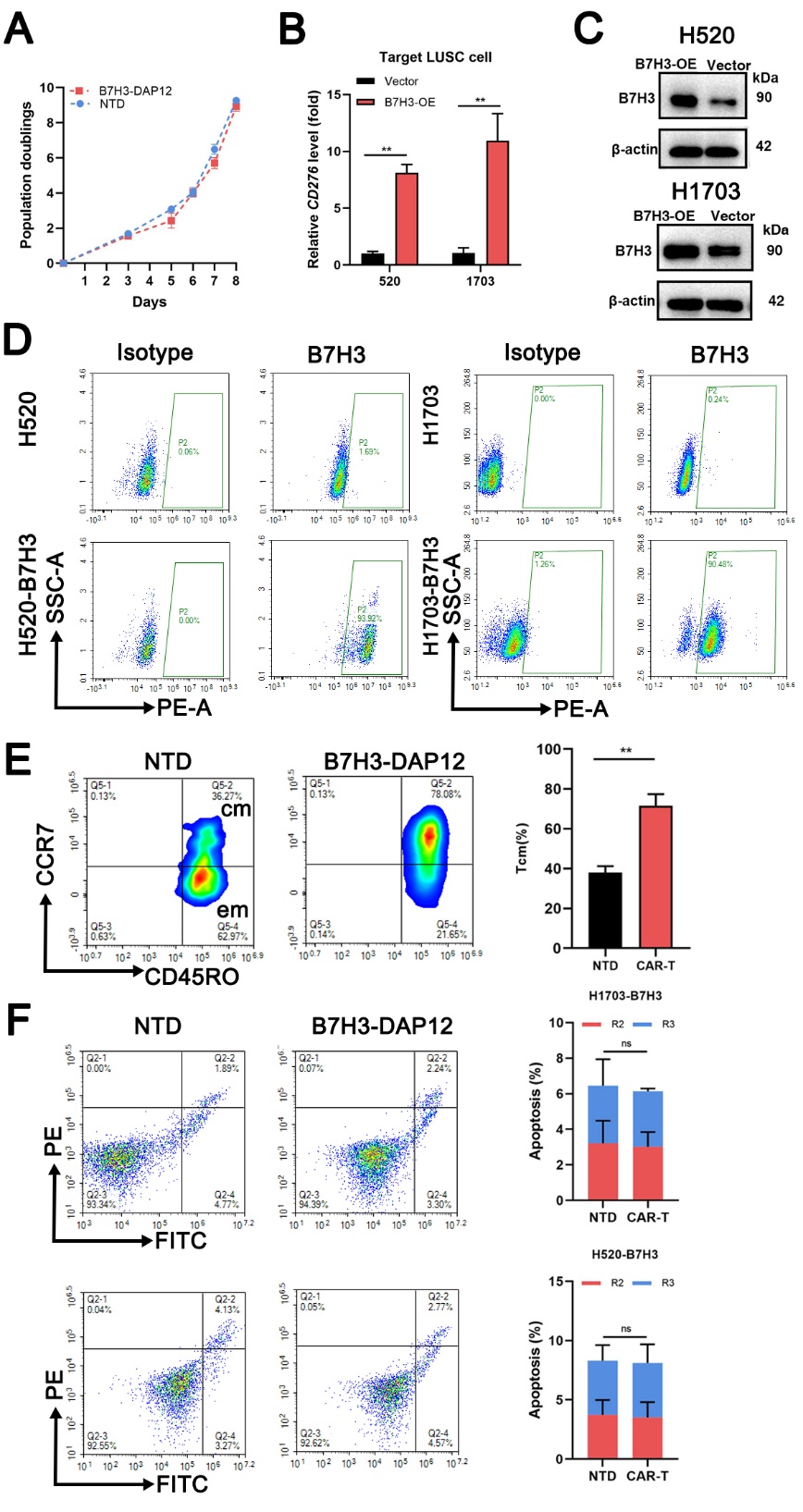


**SFig.2** **A.** Changes of the number of CAR-T per day after lentiviral infection with NTD as a control. **B-D.** B7H3 expression in H520 and H1703 infected with control or B7H3-overexpression lentivirus shown by qRT-PCR, WB, and flow cytometry. **E.** Tcm subsets of B7H3-DAP12-CAR-T or NTD at day8 shown by flow cytometry. **F.** Apoptosis rates of B7H3-DAP12-CAR-T or NTD co-cultured with target cells at E:T = 2:1 for 48h shown by flow cytometry. **P* < 0.05; ** *P* < 0.01; *** *P* < 0.001. ns, not significant. Variables are presented as mean ± SD.


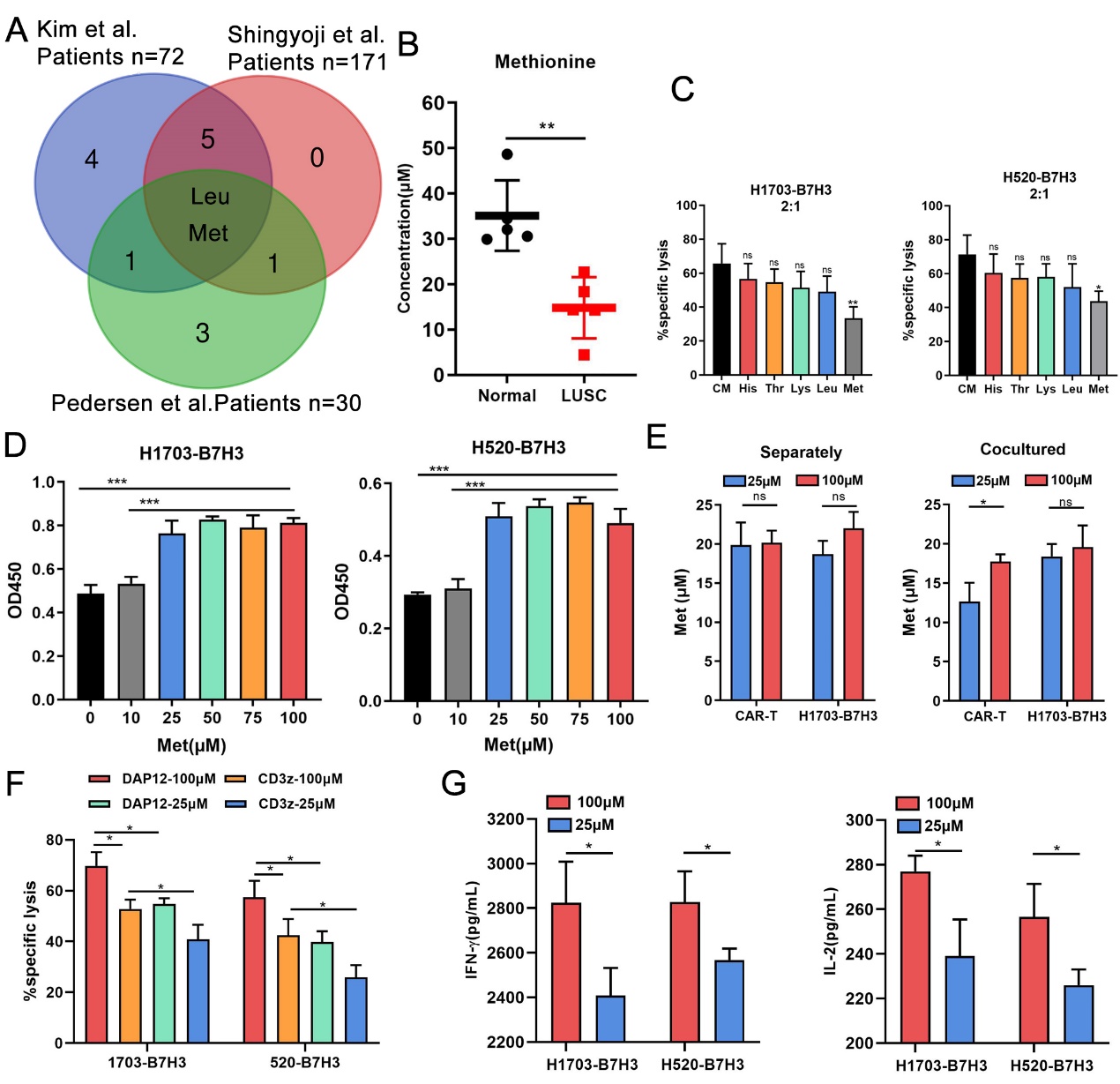


**SFig.3** **A.** Differentially expressed amino acids in three cohorts of lung cancer patients (n=72, 171, and 30, respectively). **B.** Met discrepancies between patients with LUSC and healthy individuals detected by LCMS (n=5 per group). **C.** Cytotoxicity of B7H3-DAP12-CAR-T co-cultured with 1×10^4^ LUSC cells (E:T = 2:1) for 24h in RPMI-1640 with different concentrations of amino acids (His=24µM, Thr=42µM, Lys=54µM, Leu=95µM, or Met=25µM, respectively) shown by LDH cytotoxicity assay. **D.** The growth of LUSC cells cultured in 0, 10, 25, 50, 75, and 100µM for 3 days shown by CCK8. **E.** Met concentrations in CAR-T and target cells after 2 days separately or together (E:T = 1:1) at 25 or 100µM Met shown by ELISA. **F.** Cytotoxicity of DAP12-CAR-T and CD3z-CAR-T co-cultured with 1×10^4^ LUSC cells (E:T = 2:1) for 24h at 25 or 100µM Met shown by LDH cytotoxicity assay. **G.** IL-2 and IFN-γ secreted by B7H3-CD3z-CAR-T co-cultured with target tumor cells (2 × 10^5^) at E:T = 2:1 for 48h in 25 or 100μM Met measured by ELISA. **P* < 0.05; ** *P* < 0.01; *** *P* < 0.001; ns, not significant. Variables are presented as mean ± SD.


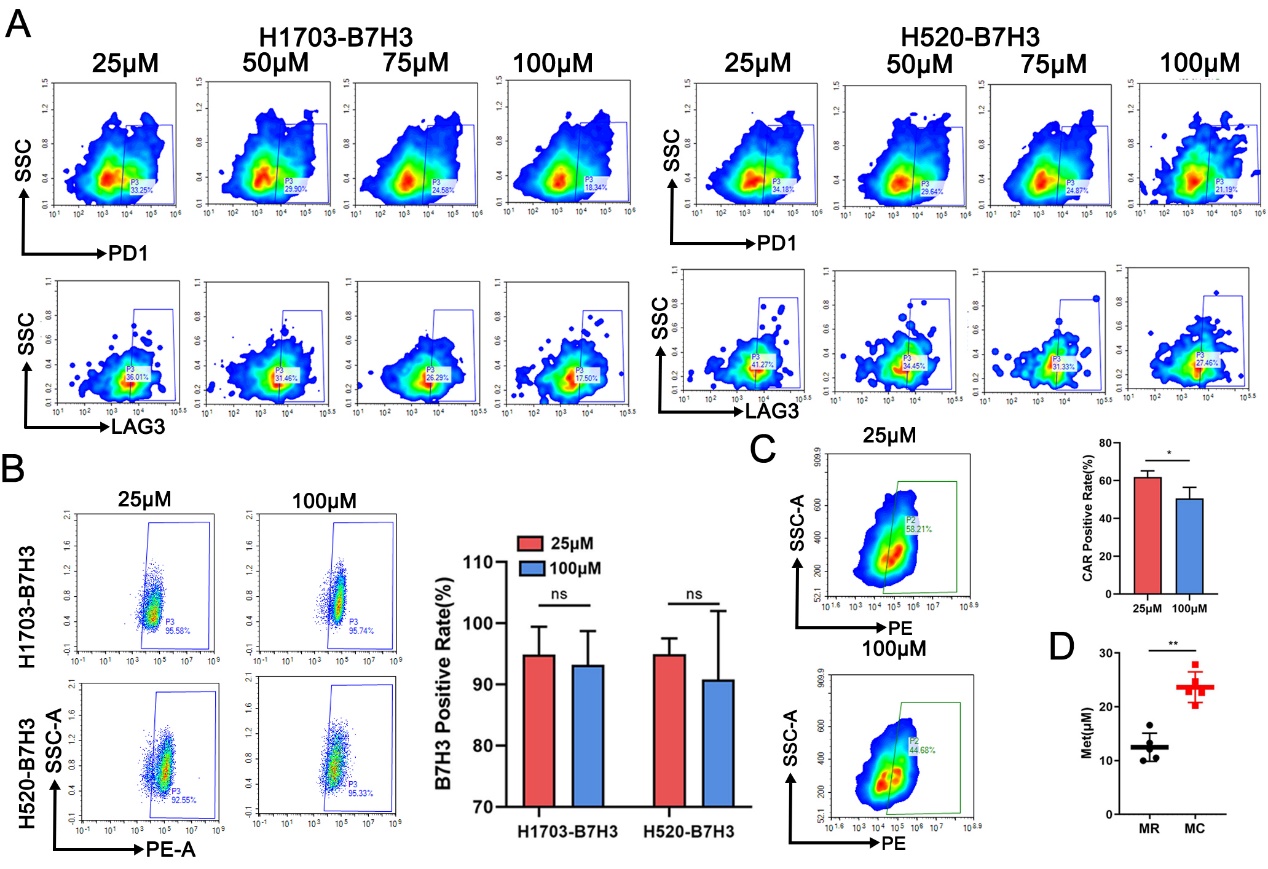


**SFig.4** **A.** Representative images of PD1 and LAG3 on B7H3-DAP12-CAR-T co-cultured with target cells at 25, 50, 75, or 100μM for 48h measured by flow cytometry. **B.** B7H3 expression on H520-B7H3 and H1703-B7H3 at 25or 100μM Met shown by flow cytometry. **C.** CAR expression on B7H3-DAP12-CAR-T at 25or 100μM Met shown by flow cytometry. **D.** Met concentration in tumors of mice fed by MR or NC feeds detected by ELISA. **P* < 0.05; ** *P* < 0.01; *** *P* < 0.001; ns, not significant. Variables are presented as mean ± SD.


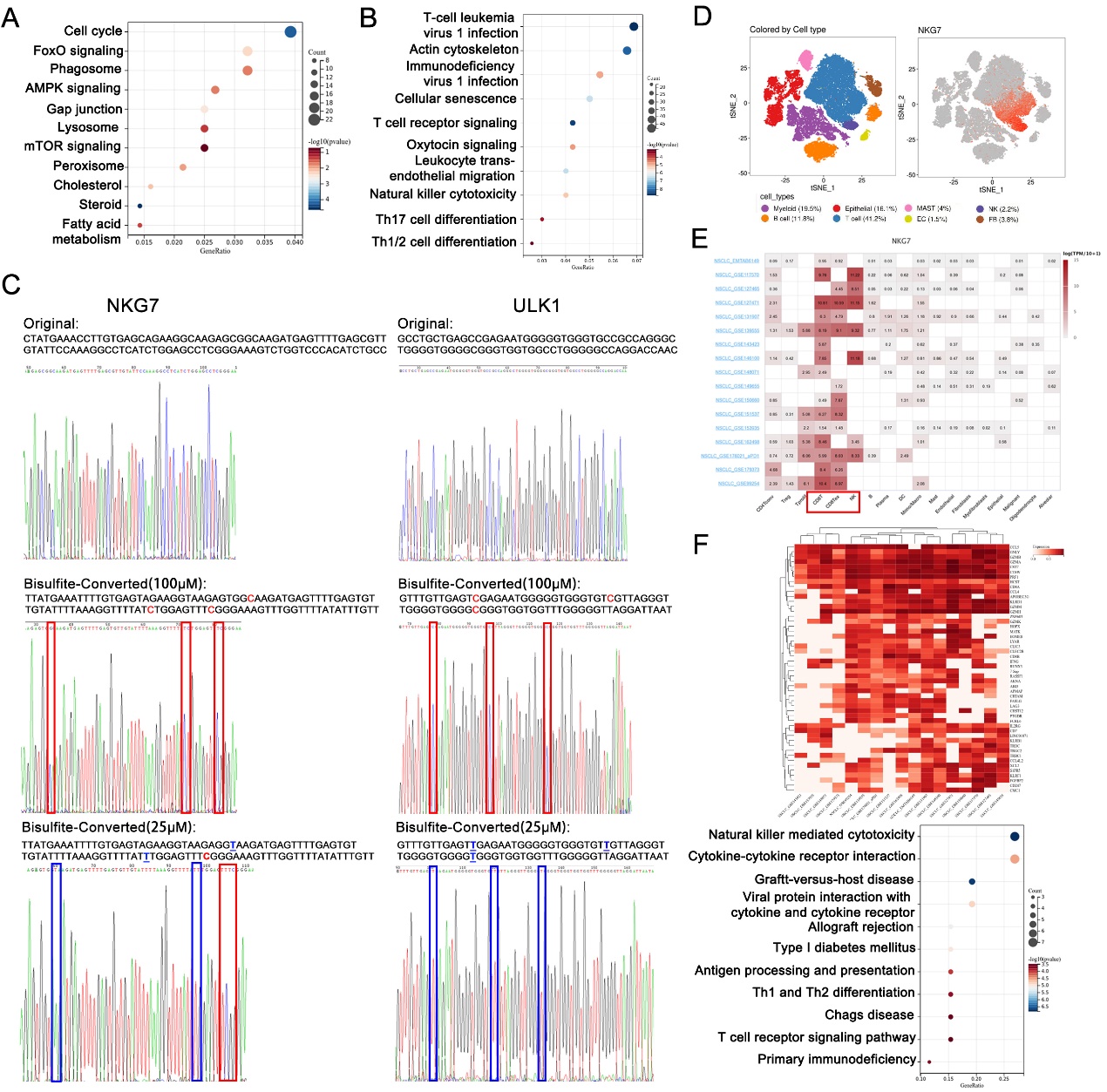


**SFig.5**. **A-B.** KEEG analysis of expression downregulated genes (n = 1538) or m5C downregulated genes (n = 1748) in CAR-T co-cultured with 1703-B7H3 (E:T = 1:1) at 25μM Met compared to those at 100μM Met. **C.** m5C sites in NKG7 and ULK1 mRNA in CAR-T co-cultured with H1703-B7H3 (E:T = 1:1) at 25 or 100μM Met detected by Sanger sequencing. mRNA without bisulfite conversion served as the reference. Red C: m5C; Blue T: converted T. **D.** Distribution of NKG7-enriched cells shown by the published single-cell sequencing. **E.** Distribution of NKG7 in different cell lines in multiple NSCLC samples shown by the database TISCH2. **F.** Heatmap of the top 50 genes correlated with NKG7 expression in TISCH2. KEEG analysis of signaling pathways containing top 50 genes correlated with NKG7 expression.


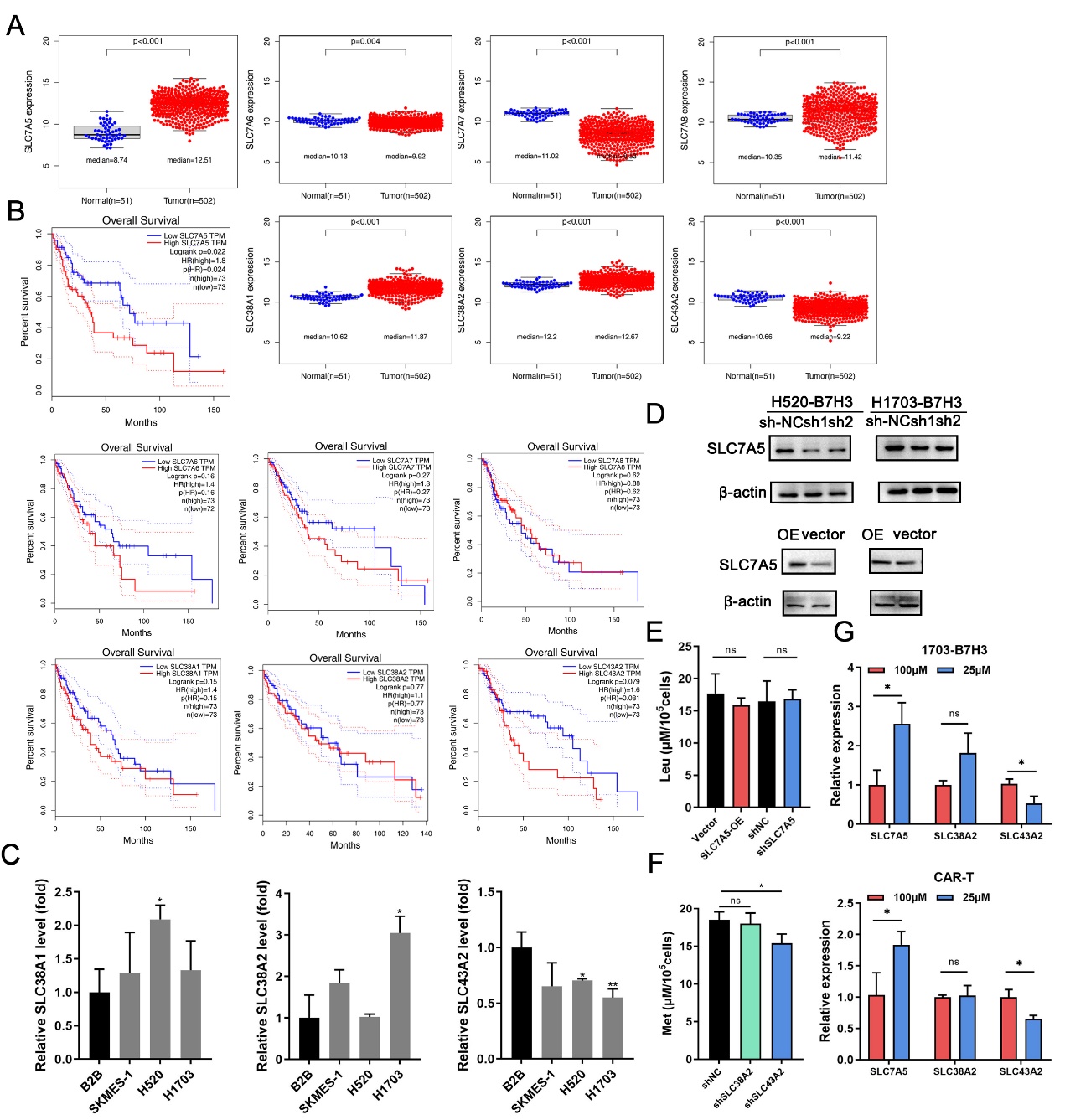


**SFig.6**. **A.** Expression of SLC7A5, SLC7A6, SLC7A7, SLC7A8, SLC38A1, SLC38A2, and SLC43A2 in LUSC (n=502) and normal adjacent tissues for (n=51) based on TCGA. **B.** Overall survival K-M plots of LUSC patients with the top 20% expression or with the bottom 20% expression of SLC7A5, SLC7A6, SLC7A7, SLC7A8, SLC38A1, SLC38A2, and SLC43A2 based on TCGA. **C.** Expression of SLC38A1, SLC38A2, and SLC43A2 in LUSC cell lines and B2B shown by qRT-PCR. **D.** Expression of SLC7A5 in LUSC cell lines with SLC7A5 up- or down-regulated shown by WB. **E.** After culturing for 2 days, Leu concentration in supernatant of 1× 10^5^ 1703-B7H3 cells with SLC7A5 up- or down-regulated detected by ELISA. **F**. After culturing for 2 days, Met concentration in supernatant of 1× 10^5^ 1703-B7H3 cells with SLC38A2 or SLC43A2 downregulated detected by ELISA. **G.** SLC7A5, SLC38A2, SLC43A2 expression in 1703-B7H3 and CAR-T after co-culturing (E:T = 2:1) for 48h in 25 or 100μM Met shown by qRT-PCR. **P* < 0.05; ** *P* < 0.01; *** *P* < 0.001; ns, not significant. Variables are presented as mean ± SD.


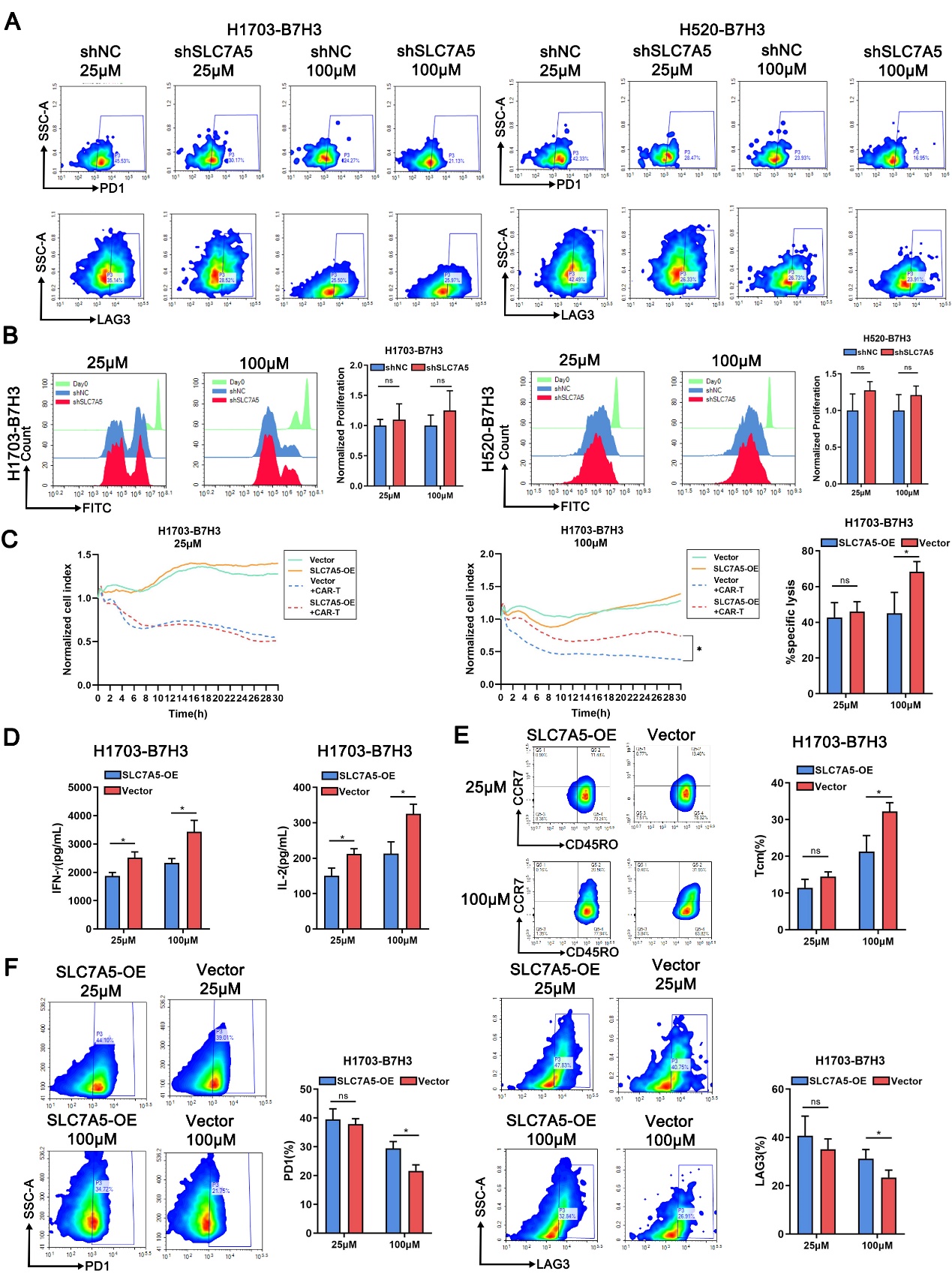


**SFig.7** **A.** Representative images of PD1 and LAG3 expression on B7H3-DAP12-CAR-T co-cultured with normal or SLC7A5 downregulated target cells at 25 or 100μM Met for 48h measured by flow cytometry. **B.** Proliferation of CFSE labelled B7H3-DAP12-CAR-T co-cultured with normal or SLC7A5 downregulated target cells at E:T = 1:1 at 25 or 100μM Met for 3 days shown by flow cytometry. **C.** Normalized cell index of B7H3-DAP12-CAR-T co-cultured with normal or SLC7A5 upregulated target cells (E:T =0:1 and 2:1) in 25 or 100μM Met within 30h. **D.** IL-2 and IFN-γ secreted by B7H3-DAP12-CAR-T co-cultured with normal or SLC7A5 upregulated target cells (2 × 10^5^) at E:T = 2:1 for 48 h in 25 or 100μM Met measured by ELISA. **E.** Tcm subsets of B7H3-DAP12-CAR-T co-cultured with normal or SLC7A5 upregulated target cells at E:T = 2:1 for 48h at 25 or 100μM shown by flow cytometry. **F.** Exhaustion markers (PD1 and LAG3) on B7H3-DAP12-CAR-T co-cultured with normal or SLC7A5 upregulated target cells at 25 or 100μM for 48h measured by flow cytometry. **P* < 0.05; ** *P* < 0.01; *** *P* < 0.001; ns, not significant. Variables are presented as mean ± SD.


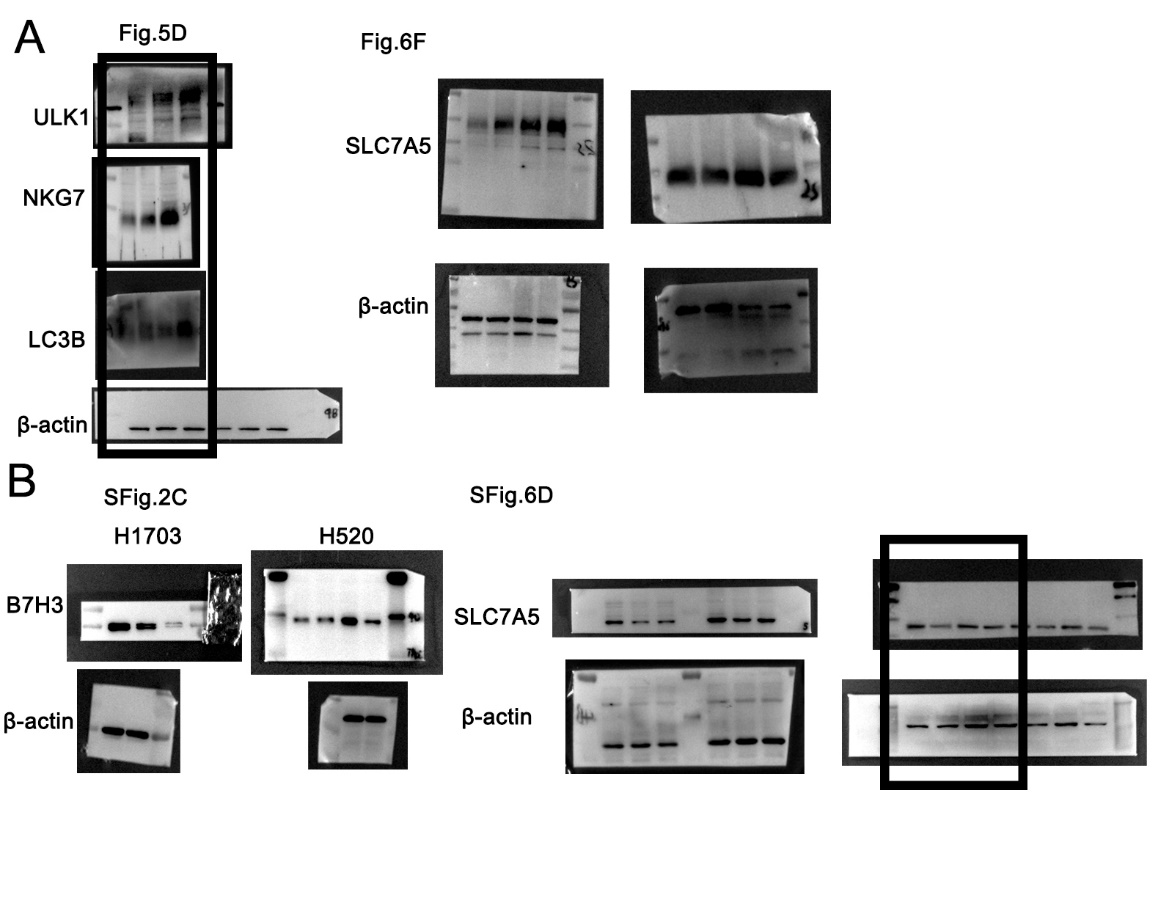


**SFig.8** **A-B.** Unprocessed scans of western blot analysis. Some immunoblotting assays membranes are cut into pieces to incubate with different antibodies. Hence, the raw images of these membranes are of small size.

**Supplementary** **Tables**

**Table S1.** Primers used for qPCR, amplification, and shRNA.

**Table S2.** Clinical information of 48 patients with LUSC.

**Table S3.** Amino acids discrepancies between patients with LUSC and healthy individuals detected by liquid chromatograph mass spectrometer.

**Table S4.** Genes significantly downregulated at 25μM Met concentration (n = 1538).

**Table S5.** Genes with significantly downregulated m5C at 25μM Met concentration (n = 1748).

**Table S6.** Immune and cell infiltration related genes (n = 761).
