# Supplementary figures and images for: Effects of methionine deficiency on B7H3-DAP12-CAR-T cells in the treatment of lung squamous cell carcinoma
Source: Cell Death Dis. 2024 Jan 5;15(1):12. doi: 10.1038/s41419-023-06376-w (PMC10770166; doi:10.1038/s41419-023-06376-w)

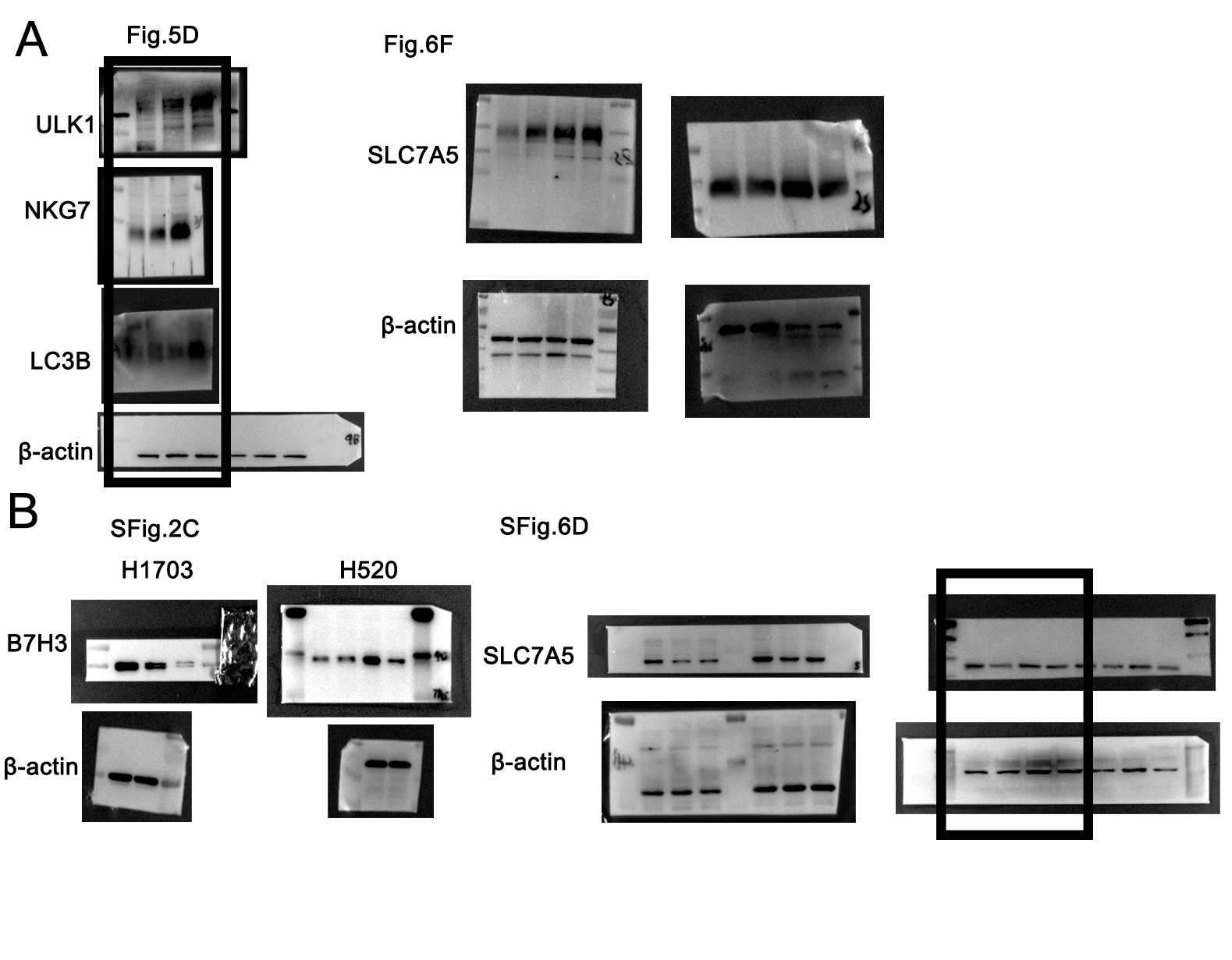

Supplement: Supplementary file 9 — Western Blots [file 41419_2023_6376_MOESM9_ESM.jpg]
